# CsrA and its regulators control the time-point of ColicinE2 release in *Escherichia coli*

**DOI:** 10.1038/s41598-018-24699-z

**Published:** 2018-04-25

**Authors:** Alexandra Götz, Matthias Lechner, Andreas Mader, Benedikt von Bronk, Erwin Frey, Madeleine Opitz

**Affiliations:** 1 Faculty of Physics and Center for NanoScience, Ludwig-Maximilians-Universität München, Geschwister-Scholl-Platz 1, D-80539 Munich, Germany; 2 Arnold-Sommerfeld-Center for Theoretical Physics and Center for NanoScience, Faculty of Physics, Ludwig-Maximilians-Universität München, Theresienstrasse 37, D-80333 Munich, Germany

## Abstract

The bacterial SOS response is a cellular reaction to DNA damage, that, among other actions, triggers the expression of colicin - toxic bacteriocins in *Escherichia coli* that are released to kill close relatives competing for resources. However, it is largely unknown, how the complex network regulating toxin expression controls the time-point of toxin release to prevent premature release of inefficient protein concentrations. Here, we study how different regulatory mechanisms affect production and release of the bacteriocin ColicinE2 in *Escherichia coli*. Combining experimental and theoretical approaches, we demonstrate that the global carbon storage regulator CsrA controls the duration of the delay between toxin production and release and emphasize the importance of CsrA sequestering elements for the timing of ColicinE2 release. In particular, we show that ssDNA originating from rolling-circle replication of the toxin-producing plasmid represents a yet unknown additional CsrA sequestering element, which is essential in the ColicinE2-producing strain to enable toxin release by reducing the amount of free CsrA molecules in the bacterial cell. Taken together, our findings show that CsrA times ColicinE2 release and reveal a dual function for CsrA as an ssDNA and mRNA-binding protein, introducing ssDNA as an important post-transcriptional gene regulatory element.

## Introduction

**Figure 1:**
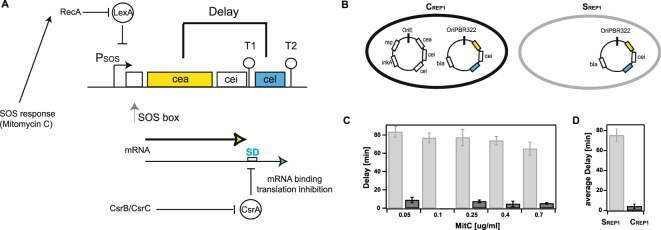
Time between toxin production and release. (**A**) The regulatory network controlling the delay between ColicinE2 production (*cea*) and release (*cel*) in C_WT_. The operon expresses the genes *cea* (ColicinE2), *cei* (immunity protein) and *cel* (protein inducing cell lysis). In the reporter plasmid, the genes *cea* and *cel* are replaced by genes encoding the fluorescent proteins YFP and CFP, respectively. The transcriptional repressor LexA inhibits expression of the operon. Cell stress causes RecA-mediated auto-cleavage of LexA dimers. Subsequently, a short *cea-cei* and a long *cea-cei-cel* mRNA are produced (T1, T2 = transcriptional terminators). Expression of *cel* is further regulated post-transcriptionally by binding of CsrA to the Shine-Dalgarno sequence (SD, depicted in blue) upstream to the *cel* gene. CsrA itself is regulated by two sRNAs, CsrB and CsrC. (**B**) Plasmids present in the two reporter strains C_REP1_ and S_REP1_. The C_REP1_ strain carries both the reporter plasmid pMO3 and pColE2-P9. The S_REP1_ strain carries only the reporter plasmid. (**C**) Dependence of the delay between ColicinE2 production and release by C_REP1_ (black) and S_REP1_ (grey) on the level of external stress (MitC concentration). (**D**) Average delay between ColicinE2 production and release by C_REP1_ (black) and S_REP1_ strains (grey). Error bars depict the standard error of the mean (SEM).

Many pathogenic bacteria outcompete close relatives by the secretion of toxic bacteriocins^1–3^, thereby increasing their own ability to dominate bacterial populations and thus increase their potential to infect a human host^3,4^. The well studied *Escherichia coli* ColicinE2 system^5–8^ represents a paradigmatic model for the study of regulatory mechanisms relevant for toxin production. Here, transcriptional and post-transcriptional regulation mechanisms control ColicinE2 expression^9^. However, it is largely not known how this regulatory network (Fig. 1A) times ColicinE2 production and release, to ensure the production of effective toxin concentrations (*cea* gene expression) and to prevent premature toxin release (*cel* gene expression) in the wild-type strain C_WT_.

Bacteriocins, including ColicinE2, are plasmid encoded and heterogeneously expressed^10–13^ from operons under the control of an SOS promoter^9,14^ in response to external stresses. The ColicinE2 operon consists of three genes: *cea* (the colicin activity gene), *cei* (the immunity gene) and *cel* (the lysis gene) (Fig. 1A). Upon induction of the SOS response, RecA induces autocleavage of LexA dimers, which permits the production of two mRNAs: the ‘short’ transcript including *cea* and *cei*, and the ‘long’ one comprising all three genes^9^. Co-expression of the genes *cea* and *cei* is necessary, since the immunity protein ensures that the colicin remains inactive for as long as the colicin-immunity protein complex is present within the cell. This complex^15^ is secreted upon *cel* gene expression^16^, which leads to the death of the bacterial cell, secreting the colicin. Translation of the *cel* gene is regulated post-transcriptionally by the mRNA binding protein CsrA^17,18^. The abundance of CsrA is further regulated by the two CsrA binding sRNAs CsrB and CsrC^19^.

Although the gene regulatory network expressing ColicinE2 (Fig. 1A) is well understood, the exact mechanism of ColicinE2 release has not been fully elucidated^9,20^. Pugsley *et al*.^16,21^ provided evidence that the lysis protein, a small lipoprotein, encoded by the *cel* gene of the ColicinE2 operon, induces the permeability of the cell envelope, thus enabling toxin release. However, the timing of lysis protein synthesis and the dynamics of ColicinE2 release are fairly unknown. In addition, quantitative investigations of Colicin expression dynamics are just emerging^10,12,13^, but urgently needed to examine the mechanisms that control the fraction of cells producing and releasing a toxin, and determine the time-point of toxin release. In this study, we investigated how different regulatory mechanisms affect the dynamics of ColicinE2 production and release in *E. coli*. We found that the global carbon storage regulator CsrA, which blocks *cel* gene translation^9,17^, controls the time - point of ColicinE2 release. The amount of free CsrA molecules is thereby dependent on several CsrA sequestering elements. Using different reporter strains, we demonstrate that the copy number of reporter plasmids carrying a CsrA binding site, highly affects the duration of the delay between toxin production and release. Furthermore, we introduce ssDNA, an intermediate of autonomous pColE2-P9 plasmid replication, as an additional - previously unknown - CsrA sequestering element, and report how its presence affects the time-point of ColicinE2 release.

## Material and Methods

### Creation of bacterial strains used in this study

All strains used in this study are listed in Table 1 and in the SI (Table S2). The strain C_WT_ represents the original wild-type strain, which carries the toxin-producing plasmid pColE2-P9. The C_REP1_ strain and the S_REP1_ strain (EMO3-C and EMO3-S, respectively) were constructed as described in Mader *et al*.^13^. Both strains carry the double reporter plasmid pMO3^13^. This multi-copy reporter plasmid enabled us to clearly distinguish toxin-expressing cells from cells that did not produce and release the toxin or only at basal levels. Furthermore, pMO3 harbours the entire ColicinE2 operon, in which the genes *cea* and *cel* have been replaced by genes coding for the fluorescence proteins (FP) mVenus (YFP) and mCerulean (CFP), respectively (Fig. 1). Hence, this plasmid retains all regulatory sequences relevant for the binding of LexA to the SOS box of the ColicinE2 operon, and of CsrA to the Shine-Dalgarno sequence (SD) on the resulting long mRNA. Besides the reporter plasmid pMO3, the C_REP1_ strain carries the toxin-producing plasmid pColE2-P9 (Table 1). To investigate the role of specific regulatory elements on the duration of the delay between *cea* and *cel* expression, all mutant strains used in this study are derived from S_REP1_. Construction of these mutant strains is described in the following.

**Table 1:** Wild-type and mutant strains used in this study.

Bacterial strain	Genetic modification/information	Expected Effect	pColE2-P9	Reporter plasmid	ssDNA
C_WT_	Wild-type strain		~20	—	+
C_REP1_	Reporter strain		~20	pMO3 ~ 55	+
S_REP1_	Reporter strain		—	pMO3 ~ 55	−
LexA1	Base exchange AT-to-TA in the first SOS box on pMO3	Stronger LexA binding, with HI factor of 8.6^35,44^	—	pMO4 ~ 55	−
LexA2	Base exchange of CTG-to-CCC in the first SOS box on pMO3	Weaker LexA binding, with HI index of 21.13^35,44^	—	pMO5 ~ 55	−
CsrA1	Mutation GTC to TGT in the second CsrA binding site in the SD sequence of *cel*	Stronger CsrA binding^37^	—	pMO6 ~ 55	−
CsrA2	Mutation AC to TT in the second CsrA binding site in the SD sequence of *cel*	Weaker CsrA binding^37,45^	—	pMO7 ~ 55	−
CsrB	In-frame replacement of CsrB with kanamycin resistance	No CsrB	—	pMO3 ~ 55	−
CsrC	In-frame replacement of CsrC with kanamycin resistance	No CsrC	—	pMO3 ~ 55	−
CsrBC	In-frame replacement of CsrC by kanamycin resistance and of CsrB by chloramphenicol resistance cassette	No CsrB and CsrC	—	pMO3 ~ 55	−
C_REP2_	Same as C_REP1_, only the origin of replication on pMO3 has been changed to p15A	Lower copy number of reporter plasmid^22^	~20	pMO8 ~ 13	+
S_REP2_	Same as S_REP1_, only the origin of replication on pMO3 has been changed to p15A	Lower copy number of reporter plasmid^22^	—	pMO8 ~ 13	−

The table summarizes the genetic details and modifications of each strain, states the resulting effect, lists the plasmids present in each strain and reports the copy number of the particular plasmid (pColE2-P9 or reporter plasmid) as well as the presence of ssDNA in the respective strain. Further strain details can be found in Table S2 and sequence specifications in Table S4.

To investigate the impact of the transcriptional repressor LexA on Colicin E2 expression, we created two LexA mutants (Tables 1, S2) by altering the LexA binding site on the pMO3 reporter plasmid using site-directed mutagenesis with the Quick ChangeII kit (Agilent Technologies). LexA1 was created using primer pair P1/P2 (Table S3), introducing the base exchange AT-to-TA on pMO3. The resulting plasmid was named pMO4 (Table S4**)**. LexA2 was created using the primer pair P3/P4 leading to the base exchange of CTG-to-CCC in the first SOS box on pMO3. The resulting plasmid was named pMO5 **(**Table S4**)**.

To analyze the post-transcriptional impact of the mRNA-binding protein CsrA on ColicinE2 expression, we altered the CsrA binding site on pMO3, again using site-directed mutagenesis. In the first mutant strain (CsrA1, Tables 1, S2), we introduced a mutation (GTC to TGT) in the second CsrA binding site **(**Table S4**)** within the SD sequence of the *cel/cfp* gene on pMO3 using the primers P5 and P6 (Table S3), generating the plasmid pMO6 (Table S4). In the second mutant (CsrA2, Tables 1, S2), the primer pair P7/P8 (Table S3) was used to alter AC to TT in the second CsrA binding site in the SD sequence of the *cel/cfp* gene on pMO3, thus inhibiting formation of the second mRNA hairpin. The resulting plasmid was named pMO7 **(**Table S4**)**.

To understand the roles of the sRNAs CsrB and CsrC in CsrA sequestration and consequently in ColicinE2 expression, single and double knock-out mutants for these sRNAs were created using the Quick&Easy *E.coli* Gene Deletion Kit Nr.6 (Gene Bridges, Heidelberg, Germany). The gene coding for the sRNA CsrB was replaced in strain BZB 1011 with a kanamycin resistance cassette using the primer pair P9/P10 **(**Table S3**)**. The resulting strain was named BZB 1011::CsrB, and the reporter plasmid pMO3 was transformed into this strain to produce EMO3::CsrB (CsrB) (SI). The single knock-out mutant of CsrC was created in a similar manner (primer P11/P12, Table S3), and was also used for the double sRNA knock-out. Here, the genomic region coding for the sRNA CsrC was replaced by a kanamycin resistance cassette. In next step, the primers P9 and P10 **(**Table S3**)** were used to replace the CsrB gene with a chloramphenicol resistance cassette. This strain was named BZB 1011::CsrB/C, and it too was transformed with the plasmid pMO3 to generate EMO3::CsrB/C (CsrB/C) (SI).

### Creation of C_REP2_ and S_REP2_

To reduce the plasmid copy number of pMO3, we exchanged the ORI of pMO3 by the p15A ORI that has a copy number of 14–16^22^. We chose this ORI, as plasmid replication from this ORI does not require additional regulatory elements, which is e.g. the case for the autonomous replication of pColE2-P9 (see SI for details on pColE2-P9 replication).

The reporter plasmid of the X_REP2_ strains was created by PCR of the plasmid pMO3 with the primer P25 and P26 (Table S3**)**, which delete the ORI of the pMO3 plasmid. The new ORI p15A was replicated via PCR using primer P27 and P28 from the Vector pZA11MCS (EXPRESSYS). After gel purification of the vector, both, the vector and the ORI p15A PCR fragment were cut with the enzymes SalI-HF and SphI-HF (NEB) and ligated in a 1:5 ratio of vector:insert using an ElectroLigase® (NEB). The resulting plasmid pMO8 was transformed into BZB 1011 and C_WT,_ respectively, creating the reporter strains S_REP2_ and C_REP2_ (Table 1). To verify the copy number of the pMO3 and pMO8 plasmids in the S_REP1_ and S_REP2_ strains, respectively, the bacteria were grown in M63 medium with antibiotic over night at 37 °C and 300 rpm. The cultures were then diluted to OD600 in an equal volume and the plasmids were purified using the QIAprep Spin Miniprep Kit (Qiagen). The concentration of the DNA was measured using the NANODROP 1000 instrument (ThermoScientific). This lead to a copy number of 55 ± 11 and 13 ± 4 plasmids per cell for the S_REP1_ and S_REP2_ strain_,_ respectively, after correction for genomic DNA and nucleotides by subtracting the background signal of similarly prepared BZB1011 cells (Table S2, the S strain wild-type that does not carry any reporter plasmid). However, the obtained copy numbers represent estimations, as small amounts of genomic DNA, or other DNA fragments may be included in the extracted samples.

### Fluorescence microscopy

**Figure 2:**
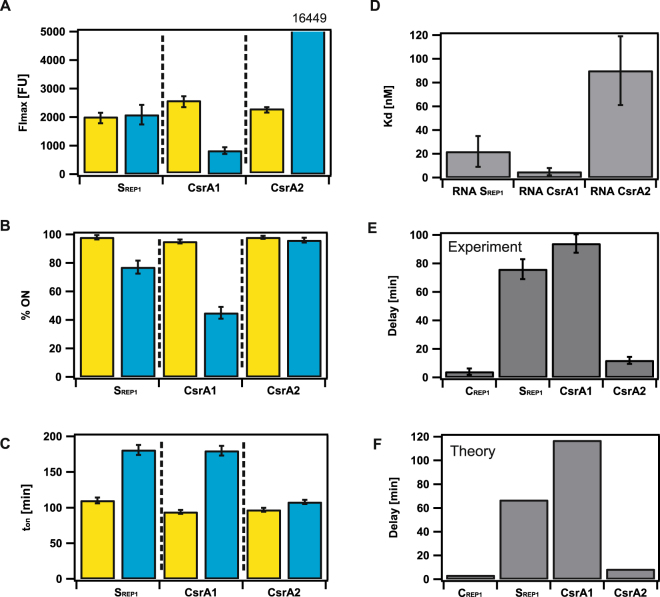
CsrA regulates the delay between ColicinE2 production (*cea*) and release (*cel*). (**A**–**C**) Yellow: *cea* gene expression, blue *cel* gene expression of cells expressing the ColicinE2 operon in the S_REP1_ strain in comparison to mutant strains CsrA1 and CsrA2. Error bars depict the standard error of the mean (SEM). (**A**) Maximal fluorescence intensity, (**B**) Cumulative fraction of cells expressing the ColicinE2 operon, (**C**) T_ON_ times for *cea* and *cel* gene expression. (**D**) Dissociation constants (K_d_) for the binding of CsrA to various RNA oligos (**SI**). (**E**) Experimentally observed *cea-cel* delay. (**F**) Theoretically determined mean *cea-cel* delay. C_REP1_ (k_M_ = 0.007), S_REP1_ (k_M_ = 0.007), CsrA1 (k_M_ = 0.0125), CsrA2 (k_M_ = 0.0018). k_M_ is the theoretical binding rate constant for CsrA binding to the long mRNA.

Bacteria were grown overnight at 37 °C in M63 minimal medium supplemented with 0.5% glycerol as a carbon source, and with 100 µg/ml ampicillin (Carl Roth, Germany) if required. Overnight cultures were diluted to an OD_600_ of 0.05 and grown to an OD_600_ of 0.2, which represents the beginning of the exponential growth phase. Aliquots (50 µl) of these cultures were allowed to attach to poly-L-lysine (BIOCHROM, Berlin)-coated Ibidi µ-slides VI^0.4^ (Ibidi GmbH, Munich) for 7.5 min and rinsed to remove unattached bacteria^13^. For time-lapse experiments, slides were then transferred to an inverse microscope, Axiovert 200 M (Carl Zeiss, Germany) equipped with an Andor camera and a Zeiss EC Plan-Neofluar 100x/1.3 oil-immersion objective. A filter set with a beam splitter BS520, an excitation bandpass HC500/24 and an emission bandpass HC 542/27 was used for YFP detection. The HC filter set for CFP detection consisted of an emission filter 483/32, a beam splitter BS458 and an excitation filter 438/24. To minimize fluorescence variations deriving from day-to-day fluctuations of the excitation source, the stability of the absolute fluorescence values was verified daily using a microscope image intensity calibration kit (Invitrogen, FokalCheck™ fluorescence microscope test slide #3) and data sets were corrected accordingly. Micromanager, an open-source program (version 1.3), was used for image acquisition^23^. After the first image, the chamber was flushed with medium containing the appropriate concentration of mitomycin C (MitC, Carl Roth, Germany). Subsequently, an image was taken every 15 min over a period of 300 min. Images were analyzed using the Cell Evaluator plug-in^24^ for ImageJ. Only live cells lying within the bright-field image were considered. General data analysis was performed using IgorPRO 6.22, Matlab (R2013b) and Adobe CS5 Software. FI_max_ represents the average maximal fluorescence intensity of single cells expressing the Colicin E2 operon. To quantify the numbers of cells expressing the FPs YFP and CFP (*cea* and *cel* gene expression, respectively) a threshold level was set to distinguish expressors from non-expressors, as described earlier^13^. The resulting fraction of cells expressing either *cea* or *cel* is given as the cumulative fraction. The time-point t_ON,_ which marks the onset of the ‘ON’ state is defined as the time at which fluorescence exceeds this switching threshold. The delay time between *cea* and *cel* gene expression was then calculated as the mean of the t_ONcel_ - t_ONcea_ values for individual cells expressing both *cea* and *cel*. Upon induction with MitC, the parameters FI_max_, % ON (Fig. S1) and delay time (Fig. 1C) show only little variation with MitC concentration (0.1, 0.25 and 0.4 µg/ml). Consequently, data presented in Figs 2, and S2 represent the average values of these three MitC concentrations to allow for better comparability and to improve statistics.

### Long-term analysis of fluorescence development

To investigate the role of sRNA knock-outs on *cea* and *cel* gene expression on a longer time-scale, experiments were performed with the Fluostar Optima Plate Reader (BMG Labtech). A 500-µl aliquot of a starter culture at OD_600_ 0.2 was induced with the appropriate MitC concentration as described above. To prevent cultures from drying out, the plate was sealed with an O_2_ permeable foil. Antibiotics were added as required, ampicillin at 100 µg/ml (Carl Roth, Germany), kanamycin at 50 µg/ml (Carl Roth, Germany) and chloramphenicol at 5 µg/ml (Carl Roth, Germany). Bacterial growth (absorbance) and YFP and CFP fluorescence development (representing *cea* and *cel* expression, respectively) was followed over a period of 16 h at 37 °C, with shaking at 300 rpm.

### ssDNA accumulation and purification

**Figure 3:**
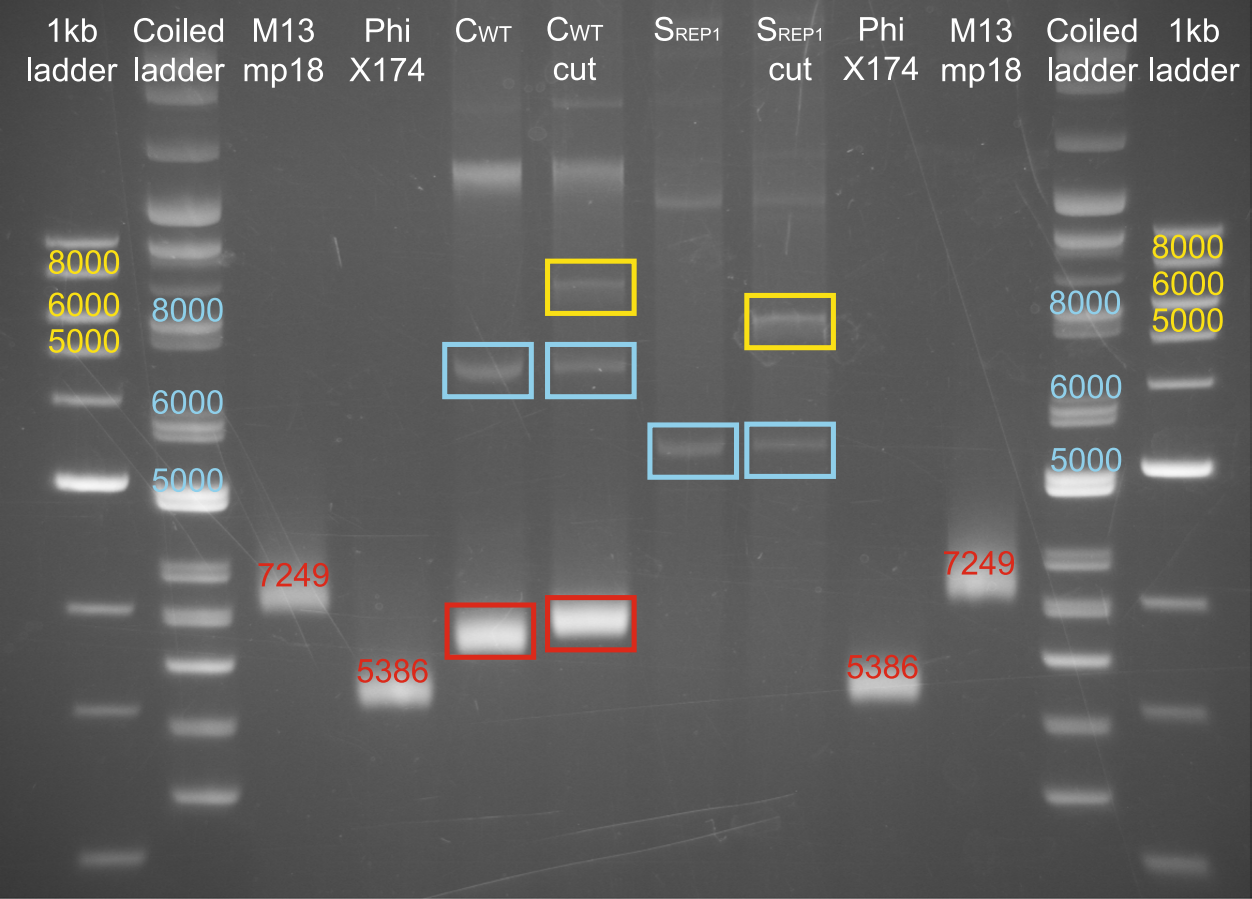
Accumulation of ssDNA in C_WT_. Agarose gel of plasmid and ssDNAs extracted from C_WT_ and S_REP1_ strains. Lanes 1–4 and 9–12 were loaded with the indicated markers: 1-kb ladder, super-coiled ladder; 7249-bp ssDNA ring (M13mp18), and 5386-bp ssDNA ring (PhiX174). Lane 5: uncleaved C_WT_ DNA showing the 6800-bp pColE2-P9 dsDNA (blue) and ssDNA (red). Lane 6: C_WT_ DNA cleaved with PvuI, showing the linearized ds pColE2-P9 plasmid (yellow). Lane 7: uncleaved S_REP1_ strain DNA showing the 5600-bp reporter plasmid (blue). Lane 8: S_REP1_ strain DNA cut with PvuI, showing the linearized reporter plasmid (yellow).

Bacterial strains were grown overnight at 37 °C in shaken cultures. The overnight cultures were induced for approximately 75 min with 0.25 µg/ml MitC. Plasmid/ssDNA extraction was performed using the Miniprep Kit (Qiagen, Germany), and 1 µg each of C_WT_ and S_REP1_ strain extracts was cleaved with PvuI (New England Biolabs (NEB), Germany). Then 300 ng of both cut and uncut C_WT_ and S_REP1_ strain extracts were applied to an 1% agarose gel, prestained with EtBr. To validate the presence of ssDNA, single-stranded circular Phi174 and M13mp18 viral DNAs (NEB, Germany) were also applied to the gel (Fig. 3).

### Sequencing and homology analysis

Sequencing of the 6757-bp pColE2-P9 was performed by MWG Eurofins Genomics (Germany) using an ABI 3730XL sequencing instrument and the sequencing primers (P13-P24) listed in the Table S3. The sequences of specific segments such as the 2640-bp ColicinE2 operon (Genbank M29885) and the Rep protein region (Genbank D30054) were verified. In all, 1754 bp were sequenced de novo, and the resulting plasmid map of the completely sequenced pColicinE2-P9 is given in Fig. S5a. The pColicinE2-P9 sequence has been deposited in GenBank (accession number KY348421). We also used the NCBI online tool BLAST to compare the sequences of genes in the pColE2-P9 plasmid with their homologues in pColE3-CA38, for which ssDNA accumulation was shown previously^25^. The sequence homologies are given in Table S1.

### Gel shift analysis

To determine the affinities of CsrA for three different RNA constructs representing the CsrA binding sites present in pMO3, pMO6 und pMO7 (SI, Table S4) we performed gel shift analysis. The N-terminal 6xHis-tagged CsrA protein used for gel shift measurements was obtained from Biozol (Germany). The folding structures of the oligos were analyzed using the Mfold web server^26^, and showed the expected double-hairpin structure that facilitates CsrA binding in the RNAs derived from pMO3, pMO6 and the ssDNA. The RNA of pMO7 however lacks this structure. The RNA was folded for 3.5 min at 85 °C in 10 mM Tris-HCl, 1 mM EDTA, 200 mM KCl, 20 mM MgCl_2_ buffer. ssDNA folding was performed under the same conditions but with 90 °C. For the gel shift analysis of RNA binding to CsrA (Fig. S5c**)**, serial 2-fold dilutions were made from a 6600 nM stock solution of CsrA (down to approximately 0.8 nM CsrA). To verify binding of CsrA to ssDNA, we performed the same gel shift analysis as done for the sRNA oligos, with sequences of 89-bp ssDNA oligos equivalent to pMO3, pMO6 and pMO7 (SI, Table S4). The samples for ssDNA gel shift analysis were prepared by doing a serial dilution starting with 33.3 µM CsrA stock solution (to a minimal CsrA concentration of approx. 4 nM CsrA). The binding reaction was performed in a buffer containing 15 mM Tris-HCl, 0.5 mM EDTA, 250 mM NaCl, 50 mM KCl, 5 mM MgCl_2_, 3.25 ng/µl yeast RNA, 4U RNase inhibitor (Ambion) and 10% glycerol buffer and incubated at 37 °C for 30 min. Gel shift measurements were performed at room temperature with precast 4–20% Mini-PROTEAN® TGX™ Precast Protein Gels (bio-rad) in Tris/Glycine Buffer (bio-rad) and run at 85 V for 1 h. Pictures of the gel shift were taken with the gel chamber ChemiDoc^TM^ MP Imaging System (bio-rad) using filters for Cy5 labels.

Using the ImageJ software, the intensity decrease in the unbound band of RNA and ssDNA for increasing CsrA concentrations was analysed for all gel shifts and plotted with IgorPro 7.04. The curves were then fitted using the following equation that was adapted from^27^.

$$FI=m-(m-b)\times [\frac{R+P+Kd-\sqrt{{(R+P+Kd)}^{2}-(4RP)}}{2R}]$$

Here FI is the measured fluorescence intensity of the RNA/ssDNA unbound band, m is the maximum FI, b the basal FI, R the used RNA/ssDNA concentration and P the CsrA concentration. The Kd for CsrA binding to ssDNA and RNA was determined on three separate days.

### Theoretical modelling

To investigate ColicinE2 expression dynamics theoretically, we extended our previous mathematical model^28^. This generalized model emulates the complex dynamical behaviour of the regulatory components and accounts for all regulatory interactions discussed in the results part of this report, including the different plasmid compositions and abundances that characterize the three strains C_REP1_, S_REP1_ and C_WT_ (Fig. S3, Table S5). As the model enables us to study various parameter values of these interactions, it gives us a controlled way to investigate the influence of the different regulatory components and mechanisms on the production and release of ColicinE2.

For the details of the theoretical analysis performed in this study we refer the reader to the SI.

### Data availability statement

All data generated or analysed during this study are included in this published article and its Supplementary Information files. The pColicinE2-P9 sequence is available at GenBank (accession number KY348421).

## Results

### The time between toxin production and release varies for different reporter strains

To study how the ColicinE2 regulatory network controls the timing of toxin expression, we investigated the dynamics of toxin production and release using a combined experimental and theoretical approach. To monitor ColicinE2 expression, we introduced an additional reporter plasmid (pMO3, Methods) into the wild-type strain C_WT_. On the reporter plasmid the genes *cea* and *cel* are replaced by sequences encoding Yellow and Cerulean fluorescence proteins (YFP and CFP), respectively (Methods), resulting in the reporter strain C_REP1_ (Table 1, Fig. 1). Using single-cell time-lapse microscopy (Methods, Fig. S1)^13^, we found that C_REP1_ expresses the *cea* and *cel* genes nearly simultaneously (Fig. 1C,D), with an insignificant delay of 4 ± 2 min.

Interestingly, a second reporter strain, S_REP1_, which only carries the reporter plasmid pMO3, but lacks the original pColE2-P9 plasmid (Tables 1, S2, Fig. S1) shows a significant delay of 75 ± 6 min between *cea* and *cel* gene expression, with a slight decrease in delay times at higher stress levels, imposed by increasing the level of the SOS response-inducing agent Mitomycin C (MitC) in the medium (Fig. 1C,D). This indicated that the original pColE2-P9 plasmid in the C_REP1_ strain, which the S_REP1_ strain lacks, contains additional regulatory elements that are responsible for the reduction of the *cea***-***cel* delay in the C_REP1_ strain.

### Effect of transcriptional regulation via LexA on the time between ColicinE2 production and release

In order to determine the key factors controlling ColicinE2 release, we analysed the roles of known transcriptional and post-transcriptional regulators of the ColicinE2 network in the strain S_REP1,_ which shows a pronounced *cea-cel* delay. We started with the role of the transcriptional repressor LexA on the duration of the delay between *cea* and *cel* gene expression. Consequently, we created two S_REP1_ strain mutants in which the LexA binding sites on the reporter plasmid had been altered, such that LexA binding was expected to increase (LexA1) or decrease (LexA2) (Methods). As expected, the maximal fluorescence intensity (FI_max_) and the fraction of ColicinE2 expressing cells (% ON) were decreased in the LexA1 mutant (Fig. S2), while the delay time did not differ significantly from that of the S_REP1_ strain, indicating that stronger LexA binding does not affect the delay between *cea* and *cel* gene expression.

In contrast to our expectations, however, the values for FI_max_ and % ON were also lower in the LexA2 mutant. Moreover, the *cea*-*cel* delay was also markedly affected, falling to 36 ± 6 min in comparison to 75 ± 6 min for the S_REP1_ strain (Fig. S2). This decrease is accounted for by the shift in the t_ON_ distribution for *cea* expression to later time-points (the time-point t_ON_ marks the onset of the ‘ON’ state, Methods, Fig. S2), indicating the absence of a post-transcriptional regulation effect.

### Post-transcriptional regulation by CsrA controls the duration of the *cea-cel* delay

A second regulatory element essential for ColicinE2 expression is the global carbon storage regulator CsrA, which inhibits *cel* expression by binding to the Shine-Dalgarno (SD) sequence present in the long mRNA (Fig. 1A). To investigate the influence of the post-transcriptional regulator CsrA on the ColicinE2 expression dynamics, we created two S_REP1_ strain mutants with altered CsrA binding sites on the pMO3 reporter plasmid (Methods, Table 1), such that CsrA binding to the SD sequence was either increased (CsrA1) or reduced (CsrA2) (Fig. 2). As expected, *cea* expression was unaffected in both mutants (Fig. 2A–C). In the CsrA1 mutant, the fraction of cells expressing the *cel* gene (% ON), and the maximal fluorescence intensity (FI_max_) of the cells in the ON state, was reduced. In contrast, FI_max_ and % ON were increased in the CsrA2 mutant (Fig. 2A,B). While the CsrA1 mutant showed an increased mean *cea-cel* delay with 94 ± 6 min, the CsrA2 mutant displayed a markedly shorter delay of 12 ± 2 min in comparison to the S_REP1_ strain (Fig. 2E). This shortening of the *cea-cel* delay is due to the earlier onset of *cel* gene expression (t_ONcel_), while the timing of *cea* gene expression (t_ONcea_) is nearly unaffected (Fig. 2C).

To elucidate the role of CsrA theoretically, we extended our previous mathematical model as described in^28^ (Methods). To show that our model is indeed valid, we employed parameters motivated by experimental studies (please see SI for details) to reproduce the delay distribution of the S_REP1_ strain (Fig. 2E,F). In agreement with the above experiment, we find that alteration of *k*_*M*_, which quantifies binding of CsrA to long mRNA resulted either in an increase (*k*_*M*_ = 0.0125) or a decrease (*k*_*M*_ = 0.0018) of the mean *cea-cel* delay in the S_REP1_ strain (Fig. 2F). Hence, our combined experimental and theoretical analysis demonstrates that CsrA is the key factor mediating the delay between toxin production and release.

### Effect of post-transcriptional regulation by the sRNAs CsrB and CsrC on the duration of the *cea-cel* delay

While CsrA directly affects the *cea-cel* delay by deferring *cel* gene expression, regulatory elements that sequester CsrA can indirectly affect the duration of the delay by controlling the abundance of the free CsrA protein. Two known CsrA-sequestering elements are the sRNAs CsrB and CsrC, which bind up to 32% of the CsrA protein in a cell^18,29–31^. Hence, deletion of these sRNAs should lead to a strong increase in CsrA abundance and consequently extend the *cea-cel* delay. We investigated the role of these sRNAs in ColicinE2 expression experimentally and first created a knock-out S_REP1_ strain mutant (Methods) lacking only the sRNA CsrB, which includes more CsrA binding sites as the second known sRNA CsrC^30^. We found that neither *cea* nor *cel* expression was significantly altered in the mutant (Fig. S2). This finding was confirmed in long-term experiments, where a decrease in FI_max_ was observed only in the very late stationary phase (Methods, Fig. S4) - which can be explained by a compensatory effect of the second sRNA CsrC^30^. Accordingly, the delay was only slightly decreased (to 68 ± 6 min) in the CsrB mutant (Fig. S2). In contrast to our expectations, a double sRNA knock-out in the S_REP1_ strain (deletion of both CsrB and CsrC) showed increased FI_max_ (Figs S2 and S4) and slightly altered % ON values for both *cea* and *cel* gene expression (Fig. S2). Interestingly, also the delay between *cea* and *cel* gene expression was significantly reduced (to 36 ± 5 min) relative to the S_REP1_ strain (Fig. S2). In addition, the onset of expression, t_ON,_ was shifted to earlier time-points for both *cea* and *cel* (Fig. S2), indicating that transcription of the entire ColicinE2 operon was prematurely induced, due to the increased availability of CsrA. A connection between CsrA abundance and the LexA-RecA network was previously described for ColicinE7 expression^32^. The reduction of the *cea*-*cel* delay was primarily due to the pronounced shift of t_ONcel_ to earlier time points in comparison to the small shift in t_ONcea_. Hence, our data imply that while deletion of a single sRNA does not affect ColicinE2 expression significantly (Figs S2 and S4), deletion of both sRNAs (CsrB and CsrC) leads to premature production and release of ColicinE2 (Fig. S2). This result points to the intervention of yet unknown regulatory mechanisms.

### Impact of plasmid copy number of reporter plasmids carrying a CsrA binding site on the duration of the *cea-cel* delay

Up to now, we have considered the impact on ColicinE2 expression of regulatory factors that are present in both S_REP1_ and C_REP1_ strains. However, it was still unclear which regulatory element deriving from the original pColicinE2-P9 plasmid (Fig. S5) reduces the *cea-cel* delay in the C_REP1_ strain. The observed differences in the *cea-cel* delay in the C_REP1_ versus S_REP1_ strain could be due to the additional 20 pColE2-P9 plasmids in the C_REP1_ strain, increasing the plasmid copy number in this strain to 75 compared to 55 in the S_REP1_ strain (Table 1, SI). As the plasmid copy number correlates with the amount of long mRNA in the presence of an SOS response, consequently, a higher amount of long mRNA able to sequester CsrA is present in C_REP1_.

To estimate the effect of the plasmid copy number/amount of long mRNA on the *cea-cel* delay, we accounted for the exact plasmid composition for each strain in the theoretical modelling (SI). As described above, for the S_REP1_ strain the theoretical analysis accurately retrieved the experimentally observed *cea-cel* delay of 67 min (Figs 2E,F and S6). For C_REP1_ the theoretical model predicted a delay of about 24 min (Fig. S6) that was significantly longer than the experimentally observed delay of 4 min (Fig. 1D). To further study the impact of the plasmid copy number on the *cea-cel* delay, we changed the origin of replication of the reporter plasmid in the way that now only ~13 reporter plasmids per cell are produced, resulting in strains C_REP2_ and S_REP2_ (Methods, Tables 1, S2). A reduction of the plasmid copy number should extend the *cea-cel* delay, as now less long mRNA is produced and consequently more free CsrA molecules are able to bind at the SD sequence of the *cel* gene. Indeed, the X_REP2_ strains with a decreased amount in total plasmid copy number show increased delay times compared to their corresponding X_REP1_ strain. For C_REP2_ with ~33 plasmid copies in total (Methods) we obtain a *cea-cel* delay of 25 ± 4 min. For S_REP2_ with ~13 plasmid copies we find a delay of >101 min, as here 67% of the cells do not express the *cel* gene and consequently do not lyse during the time frame of the experiment. Hence, the higher amount of long mRNA due to an increased plasmid copy number explains a strong reduction in the *cea-cel* delay and emphasizes that mRNAs carrying a CsrA binding site are important CsrA sequestering elements.

### The presence of ssDNA - an additional CsrA sequestering element, deriving from pColE2-P9 replication - is essential for toxin release in the wild-type strain C_WT_

In the previous paragraph, we have shown that plasmid-derived mRNAs carrying a CsrA binding site, strongly affect the delay between toxin production and release by reducing the amount of free CsrA proteins in the cell. However, this finding could not explain the discrepancy in delay times between the C and S strains, with the C strains having delay times much shorter as expected with regard to their plasmid copy number. This suggested that further regulatory or CsrA sequestering elements deriving from the pColE2-P9 plasmid must be present in the C strains, affecting the duration of the *cea-cel* delay. Consequently, we investigated genetic elements on or deriving from the pColE2-P9 plasmid that might affect ColicinE2 expression. We sequenced the entire pColE2-P9 plasmid (Methods, Fig. S5a, Genbank accession number KY348421) and performed a homology comparison of genes present on this plasmid with part of the closely related plasmid pColE3-CA38 (Table S1). As in pColE3-CA38, most genes on pColE2-P9 are involved in autonomous plasmid replication (SI), but we could not find a link between these genes and regulatory elements affecting ColicinE2 expression. It is known, that rolling-circle replication (SI) can lead to the accumulation of a ssDNA intermediate, as was shown for pColE3-CA38^25^. This ssDNA could interact with other regulatory elements affecting ColicinE2 expression, e.g. sequester the global regulatory protein CsrA, thereby further reducing the *cea-cel* delay in the C_REP_ strains. To address this hypothesis, we confirmed the accumulation of ssDNA for cells carrying the pColE2-P9 plasmid (Fig. 3) in the absence and presence of the SOS inducing agent MitC (Fig. S5b). Furthermore, we performed gel shift analysis to prove that CsrA, which is known to bind to mRNA, is also able to bind to ssDNA. Investigating the binding of CsrA to both long mRNA and ssDNA (Methods, SI), we found that CsrA binds to an RNA oligo carrying the original nucleotide sequence of the long mRNA with a K_d_ of 22 ± 13 nM, which is in good accordance with values described in literature^17,33^. Furthermore, we found that the mRNA binding protein CsrA is able to bind to ssDNA with a K_d_ of 991 ± 164 nM (Methods, Fig. S5c,d). The binding strength of CsrA to ssDNA is therefore by a factor of 45 lower as the binding strength of CsrA to sRNA (Fig. 2D). This finding, that CsrA can bind both sRNA as well as ssDNA was in accordance with previous studies revealing that CsrA possesses a KH domain^34^, a domain that is known to enable proteins to bind to mRNA as well as ssDNA^35,36^. To characterize the binding process further, in particular, to investigate if CsrA binds ssDNA at the known CsrA binding sites (Table S4) for CsrA-RNA interaction^17,37^, we studied the binding of CsrA to ssDNA with altered CsrA binding sites. We introduced the same changes in the CsrA binding site as done before for the RNA (Fig. 2D, Table S4). As seen for CsrA binding to RNA, we find that CsrA binds stronger to the sequence that should allow for stronger CsrA binding (Methods, CsrA1 sequence), and that CsrA binds weaker to the sequence that should weaken CsrA binding due to impaired formation of the second hairpin harbouring the second CsrA binding site (Fig. S5d, Methods, CsrA2 sequence). This indicates that CsrA uses the same binding motifs on the ssDNA as on the RNA - namely the GGA motif, with the neighbouring bases enabling the establishment of a hairpin structure exposing the GGA motif to allow for accurate CsrA binding^37^. However, binding of CsrA to ssDNA is by a factor of 45 less efficient than binding of CsrA to RNA. Still, ssDNA represents a relevant additional CsrA sequestering element in the C strains, as ssDNA is produced continuously during the bacterial cell cycle and accumulates in the cell to very high numbers (Figs 3, S5b). Moreover, ssDNA accumulation does not depend on the SOS response (Fig. S5b). Hence, the continuous production of ssDNA enables this regulatory element to sequester CsrA permanently. In contrast, long mRNA is produced only upon induction of the SOS response. The sRNAs CsrB and CsrC are produced during the exponential growth phase, and exhibit increased sRNA levels with entry in the stationary phase^18,30^. However, it is unclear how exactly the concentration of these sRNAs changes during the SOS response. Previous studies suggest that the sRNA level is also connected to the level of free CsrA by an indirect regulatory mechanism that involves the BarA/UvrY system^38^.

**Figure 4:**
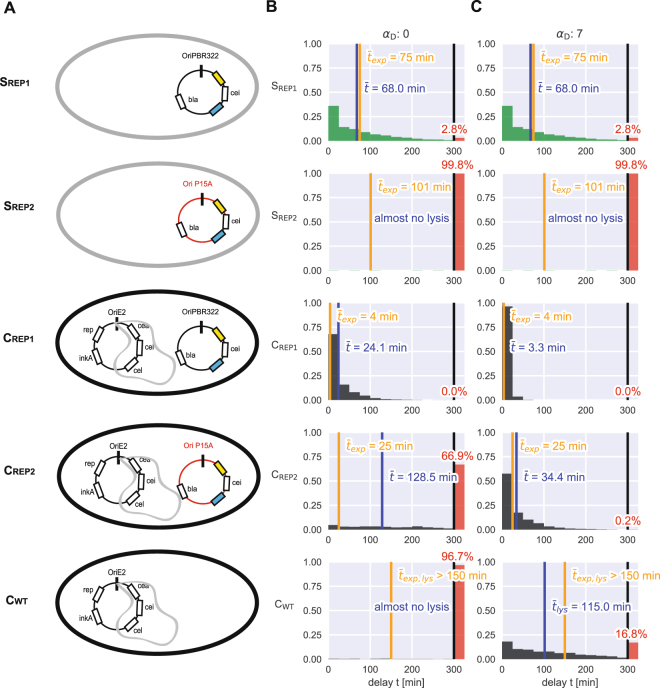
Theoretical analysis emphasizes the importance of the sequestering of CsrA by ssDNA for toxin release in C_WT_. (**A**) Plasmids and ssDNA (grey loops) present in the S_REPx_, C_REPx_ and C_WT_ strains. (**B**,**C**) Theoretical analysis of the *cea-cel* delay for all strains emphasizes the importance of ssDNA for the timing of toxin release in the colicin-producing strains (C_REPx_ and C_WT_). (**B**) no ssDNA present. (**C**) ssDNA present with α_D_ = 7. The orange line indicates the mean experimental delay for the corresponding strain, the blue line the corresponding theoretical value. The red bar on the right depicts the fraction of cells not undergoing cell lysis in the theoretical model.

To support the experimental evidence that ssDNA could, as an additional CsrA sequestering element, further reduce the *cea-cel* delay in the C_REP_ strains but also in the C_WT_ strain, we incorporated ssDNA as an additional regulatory element into the theoretical model (SI, Figs S6 and S7). Employing the experimentally measured rates and K_d_ values (including the K_d_ values for the weak CsrA binding to ssDNA, see theoretical SI section 3,) in the theory, we find that the presence of ssDNA additionally reduces the abundance of free CsrA in the C_REP_ and C_WT_ strains. Due to the increased plasmid copy number in the C_REP1_ strain and the resulting high amount of long mRNA, the presence of ssDNA can suppress the *cea-cel* delay completely (Fig. 4). For the natural C_WT_ strain carrying only the 20 pColE2-P9 plasmids, our model predicts that the *cea-cel* delay lasts approximately one hour. Furthermore, the *cea-cel* delay is broadly distributed in the wild-type strain C_WT_. Importantly, *cel* gene expression and consequently toxin release in the C_WT_ strain only occurs within the time-frame of our experimental studies if ssDNA is present (Figs 4, S8). In the absence of ssDNA, the wild-type strain C_WT_ is unable to lyse (Fig. S6) and to release the toxin. This emphasizes the importance of ssDNA for the natural colicin producing strain to enable toxin release.

### The duration of the *cea-cel* delay is controlled by CsrA and its regulators

**Figure 5:**
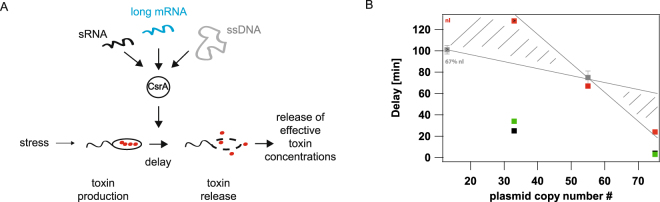
Time-point of ColicinE2 release is regulated by the global carbon storage regulator CsrA. (**A**) CsrA controls the delay between toxin production and release. This mechanism prevents premature release of ineffective toxin concentrations. CsrA abundance is regulated by several components: the long mRNA transcribed from the pColE2-P9 plasmid and the reporter plasmid pMO3 (or pMO8), the sRNAs CsrB and CsrC, and the newly discovered regulatory element ssDNA originating and accumulating from autonomous rolling circle plasmid replication. (**B**) Our experimental and theoretical data emphasize the importance of the amount of long mRNA that correlates with the plasmid copy number, as well as the presence of ssDNA as CsrA sequestering elements affecting the *cea-cel* delay. C strains are shown in black, S strains are shown in grey. The grey sketched area depicts the area of expected delay times of all strains in dependence to the plasmid copy number only. Please note that due to the fact that at low plasmid copy numbers many cells do not lyse (nl) these delay times cannot be given as an exact value, but are estimated to lie in the depicted grey area. Red dots represent the values of the theoretical analysis in the absence of ssDNA (so plasmid copy number effect only), green markers represent the theoretical values in the presence of ssDNA, which is the case for all C strains.

These results show that the delay time of a particular strain is mainly controlled by three CrsA sequestering components (Fig. 5A). First, the action of the sRNAs that are present in all strains studied in this work. Second, the amount of long mRNA that depends on the type and number of plasmid present in the particular strain (Fig. 5B, SI, Table S5) and third, the additional accumulation of ssDNA in strains carrying the pColE2-P9 plasmid (C strains, Fig. 5B). Consequently, the more CsrA sequestering elements are present within a bacterial cell at a particular time-point, the lower is the amount of free CsrA molecules. This statement is also supported by our model (SI), which shows that the delay length indeed depends on the abundance of free CsrA, and that this abundance, due to its small size of a few hundred molecules^39^, is significantly affected even by weakly binding elements like ssDNA (SI, Fig. S7).

Notably, the effect of the three CsrA sequestering elements differs due to their occurrence (SI); e.g. while long mRNA is only produced upon induction via the SOS response, sRNA production is increased when entering the stationary phase^18,30^. In contrast, ssDNA is produced independently and accumulates to high levels within the bacterial cell due to autonomous rolling circle replication in the presence and absence of an SOS response (Fig. S5b).

## Discussion

In this study, we investigated regulatory factors controlling ColicinE2 production and release in response to an SOS signal. We found that transcriptional regulation by the repressor LexA can affect the duration of the time delay between *cea* and *cel* gene expression by altering the timing of the onset of *cea* expression (t_ONcea_). However, it is difficult to determine if indeed LexA or a further transcriptional protein AsnC is causing the shift in t_ONcea_. Kamensek *et al*.^40^ report that AsnC controls the temporal induction of the ColicinE2 operon in concert with LexA. As the AsnC protein also binds within the LexA binding sites^40,41^, changes in the LexA binding site could also alter AsnC binding, thus affecting the initiation of ColicinE2 transcription. However, the observed changes in the duration of the *cea-cel* delay, which are due to transcriptional regulation, were insufficient to explain the simultaneous expression of *cea* and *cel* genes in the C_REP1_ strain. In contrast, we were able to demonstrate that a post-transcriptional regulator - the global carbon storage regulator CsrA - controls the time-point of ColicinE2 release in *Escherichia coli*. This mRNA binding protein CsrA is highly abundant in the *E. coli* cell, with 11.000–33.000 CsrA molecules in total (bound and unbound)^18^. However, Taniguchi *et al*., report that only a small fraction of a few hundred CrsA molecules per cell are freely available^39^. This indicates that CsrA sequestering elements can strongly affect the amount of free CsrA in the bacterial cell. Only recently, it was also shown that the amount of free CsrA is also affected by host cell attachment that elicits post-transcriptional regulation in enteropathogenic bacteria^42^.

Two well studied CsrA sequestering components are the sRNAs CsrB and CsrC^18,30,31^. In fact, these sRNAs are the main binding partners of CsrA, as CsrB alone can bind up to 32% of CsrA present in the bacterial cell^18^. In this study, we were able to show that besides these sRNAs, also mRNAs that carry a CsrA binding site^43^ can strongly reduce the abundance of free CsrA. Furthermore, we verified that CsrA is able to bind to ssDNA originating from autonomously replicating plasmids. Our data indicate that CsrA is thereby binding to the GGA motif exposed in the second hairpin loop present in both ssDNA and long mRNA deriving from the pColE2-P9. This demonstrates the dual role of CsrA as an mRNA and ssDNA binding protein. In addition, our study shows that in *E. coli* cells carrying autonomously replicating plasmids with a CsrA binding site, ssDNA deriving from these plasmids can serve as an additional CsrA sequestering element. Our theoretical analysis revealed that the natural colicin producing strain C_WT_ is unable to release the toxin in the absence of the ssDNA. A similar result is found if the plasmid copy number is reduced to one in comparison to the ~20 copies of the natural colicin producing plasmid pColE2-P9 (Fig. S9). This finding is in agreement with previous studies by Pugsley *et al*.^16^, stating that bacterial cells with a chromosomally expressed colicin operon did not produce sufficient ColicinE2 to be detected by SDS-PAGE of whole cell extracts. This indicates that a plasmid encoded ColicinE2 operon is preferable over a chromosomally encoded operon for two reasons: (i) the higher copy number allows for higher toxin and lysis protein concentrations and (ii) the presence of an ssDNA intermediate enables early toxin release by further reducing the amount of free CsrA in the bacterial cell. Consequently, we speculate that ssDNA accumulating in bacterial cells could play an important regulatory role in other protein-expressing networks that rely on the expression of proteins from autonomously replicating plasmids, as is the case for many bacteriocin-producing networks. With regard to the ColicinE2 system, our combined experimental and theoretical efforts allowed us to disentangle the different regulatory mechanisms affecting the delay between toxin production and release. We revealed that the interplay between CsrA and ssDNA, sRNAs and long mRNA times toxin release upon induction of the SOS response (Fig. 5). In particular, our theoretical investigations emphasized that the presence of ssDNA can enable the toxin producer to release the toxin within few hours once an SOS response has been triggered. From an evolutionary perspective, a short delay might be important for the toxin producing colony to respond quickly to changing environmental conditions and to increase its competitive success.

## Electronic supplementary material

Supplementary information

## References

[1] Bakkal S, Robinson SM, Ordonez CL, Waltz DA, Riley MA (2010). Role of bacteriocins in mediating interactions of bacterial isolates taken from cystic fibrosis patients. Microbiology.

[2] Nedialkova LP (2014). Inflammation fuels colicin Ib-dependent competition of Salmonella serovar Typhimurium and E. coli in enterobacterial blooms. PLoS Pathog.

[3] Kommineni S (2015). Bacteriocin production augments niche competition by enterococci in the mammalian gastrointestinal tract. Nature.

[4] Bashan A (2016). Universality of human microbial dynamics. Nature.

[5] Weber MF, Poxleitner G, Hebisch E, Frey E, Opitz M (2014). Chemical warfare and survival strategies in bacterial range expansions. J R Soc Interface.

[6] Reichenbach T, Mobilia M, Frey E (2007). Mobility promotes and jeopardizes biodiversity in rock-paper-scissors games. Nature.

[7] Kerr B, Riley MA, Feldman MW, Bohannan BJ (2002). Local dispersal promotes biodiversity in a real-life game of rock-paper-scissors. Nature.

[8] Kirkup BC, Riley MA (2004). Antibiotic-mediated antagonism leads to a bacterial game of rock-paper-scissors *in vivo*. Nature.

[9] Cascales E (2007). Colicin biology. Microbiol Mol Biol Rev.

[10] Kamensek S, Podlesek Z, Gillor O, Zgur-Bertok D (2010). Genes regulated by the Escherichia coli SOS repressor LexA exhibit heterogeneous expression. BMC Microbiol.

[11] Ozeki H, Stocker BA, De Margerie H (1959). Production of colicine by single bacteria. Nature.

[12] Mrak P, Podlesek Z, van Putten JP, Zgur-Bertok D (2007). Heterogeneity in expression of the Escherichia coli colicin K activity gene cka is controlled by the SOS system and stochastic factors. Mol Genet Genomics.

[13] Mader A (2015). Amount of colicin release in Escherichia coli is regulated by lysis gene expression of the colicin E2 operon. PloS one.

[14] Riley MA, Wertz JE (2002). Bacteriocins: evolution, ecology, and application. Annu Rev Microbiol.

[15] Wojdyla JA, Fleishman SJ, Baker D, Kleanthous C (2012). Structure of the ultra-high-affinity colicin E2 DNase–Im2 complex. Journal of molecular biology.

[16] Pugsley AP, Goldzahl N, Barker RM (1985). Colicin E2 production and release by Escherichia coli K12 and other Enterobacteriaceae. Journal of general microbiology.

[17] Yang TY, Sung YM, Lei GS, Romeo T, Chak KF (2010). Posttranscriptional repression of the cel gene of the ColE7 operon by the RNA-binding protein CsrA of Escherichia coli. Nucleic acids research.

[18] Gudapaty S, Suzuki K, Wang X, Babitzke P, Romeo T (2001). Regulatory interactions of Csr components: the RNA binding protein CsrA activates csrB transcription in Escherichia coli. Journal of bacteriology.

[19] Suzuki K, Babitzke P, Kushner SR, Romeo T (2006). Identification of a novel regulatory protein (CsrD) that targets the global regulatory RNAs CsrB and CsrC for degradation by RNase E. Genes Dev.

[20] James R, Kleanthous C, Moore GR (1996). The biology of E colicins: paradigms and paradoxes. Microbiology.

[21] Pugsley AP, Schwartz M (1984). Colicin E2 release: lysis, leakage or secretion? Possible role of a phospholipase. The EMBO journal.

[22] Hiszczynska-Sawicka E, Kur J (1997). Effect of Escherichia coli IHF mutations on plasmid p15A copy number. Plasmid.

[23] Edelstein, A., Amodaj, N., Hoover, K., Vale, R. & Stuurman, N. Computer control of microscopes using microManager. *Current protocols in molecular biology/edited by Frederick M. Ausubel…* [*et al*.] Chapter 14, Unit1420, 10.1002/0471142727.mb1420s92 (2010). 10.1002/0471142727.mb1420s92 PMC3065365 20890901

[24] Youssef S, Gude S, Radler JO (2011). Automated tracking in live-cell time-lapse movies. Integrative biology: quantitative biosciences from nano to macro.

[25] Morales M, Attai H, Troy K, Bermudes D (2015). Accumulation of single-stranded DNA in Escherichia coli carrying the colicin plasmid pColE3-CA38. Plasmid.

[26] Zuker M (2003). Mfold web server for nucleic acid folding and hybridization prediction. Nucleic acids research.

[27] Pagano JM, Clingman CC, Ryder SP (2011). Quantitative approaches to monitor protein-nucleic acid interactions using fluorescent probes. Rna.

[28] Lechner M, Schwarz M, Opitz M, Frey E (2016). Hierarchical Post-transcriptional Regulation of Colicin E2 Expression in Escherichia coli. PLoS Comput Biol.

[29] Babitzke P, Romeo T (2007). CsrB sRNA family: sequestration of RNA-binding regulatory proteins. Current opinion in microbiology.

[30] Weilbacher T (2003). A novel sRNA component of the carbon storage regulatory system of Escherichia coli. Mol Microbiol.

[31] Liu MY (1997). The RNA molecule CsrB binds to the global regulatory protein CsrA and antagonizes its activity in Escherichia coli. The Journal of biological chemistry.

[32] Chang HW, Yang TY, Lei GS, Chak KF (2013). A novel endogenous induction of ColE7 expression in a csrA mutant of Escherichia coli. Curr Microbiol.

[33] Baker CS (2007). CsrA inhibits translation initiation of Escherichia coli hfq by binding to a single site overlapping the Shine-Dalgarno sequence. Journal of bacteriology.

[34] Liu MY, Yang H, Romeo T (1995). The product of the pleiotropic Escherichia coli gene csrA modulates glycogen biosynthesis via effects on mRNA stability. Journal of bacteriology.

[35] Lewis LK, Harlow GR, Gregg-Jolly LA, Mount DW (1994). Identification of High Affinity Binding Sites for LexA which Define New DNA Damage-inducible Genes in Escherichia coli. Journal of molecular biology.

[36] Dickey TH, Altschuler SE, Wuttke DS (2013). Single-stranded DNA-binding proteins: multiple domains for multiple functions. Structure.

[37] Dubey AK, Baker CS, Romeo T, Babitzke P (2005). RNA sequence and secondary structure participate in high-affinity CsrA-RNA interaction. Rna.

[38] Seyll E, Van Melderen L (2013). The ribonucleoprotein Csr network. International journal of molecular sciences.

[39] Taniguchi Y (2010). Quantifying E. coli proteome and transcriptome with single-molecule sensitivity in single cells. Science.

[40] Kamensek S (2015). Silencing of DNase Colicin E8 Gene Expression by a Complex Nucleoprotein Assembly Ensures Timely Colicin Induction. PLoS Genet.

[41] Fornelos N, Browning DF, Butala M (2016). The Use and Abuse of LexA by Mobile Genetic Elements. Trends Microbiol.

[42] Katsowich N (2017). Host cell attachment elicits posttranscriptional regulation in infecting enteropathogenic bacteria. Science.

[43] Timmermans J, Van Melderen L (2010). Post-transcriptional global regulation by CsrA in bacteria. Cellular and molecular life sciences: CMLS.

[44] Fernández de Henestrosa AR (2000). Identification of additional genes belonging to the LexA regulon in Escherichia coli. Molecular Microbiology.

[45] Gutierrez P (2005). Solution structure of the carbon storage regulator protein CsrA from Escherichia coli. Journal of bacteriology.

