# Supplementary material for: CsrA and its regulators control the time-point of ColicinE2 release in Escherichia coli
Source: Sci Rep. 2018 Apr 25;8:6537. doi: 10.1038/s41598-018-24699-z (PMC5916893; doi:10.1038/s41598-018-24699-z)
Supplement: Supplementary file 1 — Supplementary information [file 41598_2018_24699_MOESM1_ESM.pdf]

Supplementary information for

Title: CsrA and its regulators control the time-point of ColicinE2 release in *Escherichia coli*

Alexandra Götz, Matthias Lechner, Andreas Mader, Benedikt von Bronk, Erwin Frey and Madeleine Opitz

Supplementary Information (experimental)

Accumulation of single-stranded DNA (ssDNA)

Recently, Morales *et al.*(1), detected the accumulation of ssDNA in *Escherichia coli* cells carrying any one of the plasmids pColE3-CA38, pColE9 and pColE5. This ssDNA originates from rolling-circle replication(1, 2). This type of replication enables an autonomous plasmid to replicate independently of the bacterial cell cycle and cell division(3). The replicons of the plasmids pColE2-P9 and pColE3-CA38 are closely related(4), indicating that asymmetric rolling-circle replication(5) of the pColE2-P9 plasmid (**Fig S5a**) could also lead to ssDNA accumulation, as shown in **Fig 3 and Fig S5b**. Furthermore, sequencing of pColE2-P9 and an analysis of its homology with pColE3-CA38 (**Table S1**) confirms the close relationship between the two plasmids. The Rep protein binds to the ColE2 *ori* to initiate rolling-circle replication(3), leading to ssDNA synthesis(2). The concentration of the Rep protein is held constant by the action of an anti-sense sRNA (the *inkA* gene product) that binds to the 5'-untranslated region of the mRNA encoding the Rep protein(6).
Please note that the reporter plasmid pMO3(7) used in this study is a derivative of pBAD24. Its replication mode(8) differs from that of pColE2-P9 and does not lead to the accumulation of ssDNA (**Fig 3**)(1).

Supplementary Methods

In the following, additional information relating to the construction of the specific mutant strains used in this study is given, together with a description of RNA and ssDNA oligos used for CsrA binding studies. **Table S2** provides an overview of all (mutant) strains used in this study. **Table S3** summarizes all primers used, including those employed in the construction of the mutant strains listed in **Table S4** and in sequencing of pColE2-P9. **Table S4** lists the base-pair changes introduced into the plasmid pMO3 that resulted in plasmids pMO4, pMO5, pMO6, pMO7, as well as the sequences of RNA and ssDNA oligos used for CsrA-binding studies as described in the **Methods** section of the main text.

Supplementary Tables

Table S1: Sequence homology of genes present on the plasmids pColE2-P9 and pColE3-CA38. Numbers in red indicate that the gene extends past the base-pair designated as position 1.

Gene	Sequence position on pColicinE2-P9	Sequence position on pColE3-CA38	Homology E2/E3
rep	3073-3792	3344-4258	97%
ssi	4084-4159	4376-4451	100%
mob	4182-4456	4475-4750	98%
tra	4636-6757,1-98	4929-7118,1-30	97%
incA	2943-3057	3214-3328	100%
colE2/E3 ori	3966-3997	4257-4289	94%

Table S2: Bacterial strains used in this study

Bacterial strain	Strain description	Genetic modification/information	Reference
BZB 1011	The S wild-type strain		(9)
C_{WT} (BZB 1011 E2C)		Carries pColE2-P9	(9)
C_{REP1} (EMO3-C)	BZB 1011 E2C pMO3	Carries pColE2-P9 and the reporter plasmid pMO3	(7)
S_{REP1} (EMO3-S)	BZB 1011 pMO3	Same as EMO3-C without the Colicin E2 plasmid	(7)
LexA1	BZB 1011 pMO4 Derivative of S_{REP1}	LexA binding sequence altered on pMO3 to achieve stronger LexA binding, resulting in pMO4	This study
LexA2	BZB 1011 pMO5 Derivative of S_{REP1}	LexA binding sequence altered on pMO3 to achieve weaker LexA binding, resulting in pMO5	This study
CsrA1	BZB 1011 pMO6 Derivative of S_{REP1}	CsrA binding sequence on pMO3 altered to achieve stronger CsrA binding, resulting in pMO6	This study
CsrA2	BZB 1011 pMO7 Derivative of S_{REP1}	CsrA binding sequence on pMO3 altered to achieve weaker CsrA binding, resulting in pMO7	This study
CsrB (EMO3::CsrB)	BZB 1011 CsrB::Kan pMO3 Derivative of S_{REP1}	CsrB::Kan, in-frame replacement of CsrB by a kanamycin resistance	This study
CsrC (EMO3::CsrC)	BZB 1011 CsrC::Kan pMO3 Derivative of S_{REP1}	CsrC::Kan, in-frame replacement of CsrC with a kanamycin resistance	This study
CsrBC (EMO3::CsrBC)	BZB 1011 CsrB::Cam CsrC::Kan pMO3 Derivative of S_{REP1}	CsrC::Kan, CsrB::Cam, in-frame replacement of CsrC by a kanamycin resistance and of CsrB by a chloramphenicol resistance cassette	This study
C_{REP2}	BZB 1011 E2C pMO8	Same as C_{REP1}, only the origin of replication on pMO3 has been changed to p15A to achieve a lower copy number of 13 copies per cell - pMO8	This study
S_{REP2}	BZB 1011 pMO8	Same as S_{REP1}, only the origin of replication on pMO3 has been changed to p15A to achieve a lower copy number of 13 copies per cell -> pMO8	This study

Table S3: Primers used in this study. Primers P1-P12 were used for construction of the mutant strains listed in **Table S2**. Primers P13-P24 were used for sequencing pColE2-P9. Primer pairs P25/P26 and P27/28 were used to create the low copy plasmid pMO8.

Name	Sequence	Purpose
P1	5´- GACGGGTACTTTTTGTACTGTACATAAAACCAGTGG - 3´	LexA1 [fwd] cloning
P2	5´- CCACTGGTTTTATGTACAGTACAAAAAGTACCCGTC- 3´	LexA1 [rev] cloning
P3	5´- GACGGGTACTTTTTGATCCCTACATAAAACCAGTGG- 3´	LexA2 [fwd] cloning
P4	5´- CCACTGGTTTTATGTAGGGATCAAAAAGTACCCGTC- 3´	LexA2 [rev] cloning
P5	5´- GGCATTCTTTCACATTAAGGAGTCGTTATG - 3´	CsrA1 [fwd] cloning
P6	5´- CATAACGACTCCTTAATGTGAAAGAATGCC- 3´	CsrA1 [rev] cloning
P7	5´- GCATTCTTTCACAACAAGGATGTGTTATGAAAAAAATAACCGG-3´	CsrA2 [fwd] cloning
P8	5´- CCGGTTATTTTTTTCATAACACATCCTTGTTGTGAAAGAATGC- 3´	CsrA2 [rev] cloning
P9	5´GTGGTCATAAAGCAACCTCAATAAGAAAAACTGCCGCGAA GGATAGCAGG AATTAACCCTCACTAAAGGGCG 3´	ΔCsrB [fwd] cloning
P10	5´TTGTCTGTAAGCGCCTTGTAAGACTTCGCGAAAAAGACGATTCTATCT TCTAATACGACTCACTATAGGGCTC 3´	ΔCsrB [rev] cloning
P11	5´ ACTGATGGCG GTTGATTGTT TGTTTAAAGCAAAGGCGTAA AGTAGCACCCAATTAACCCTCACTAAAGGGCG 3´	ΔCsrC [fwd] cloning
P12	5´GCCGTTTTATTCAGTATAGATTTGCGGCGGAATCTAACAGAAAGCAA GCATAATACGACTCACTATAGGGCTC 3´	ΔCsrC [rev] cloning
P13	5´- ACCGTATCTCCGTCATCAAC -3´	ColE2-1 [fwd] sequencing
P14	5´- CTTCCTGTGAGAACTGC -3´	ColE2-2 [fwd] sequencing
P15	5´- GTAGCGAGCGAATGAG -3´	ColE2-3 [fwd] sequencing
P16	5´- CATGATTGCCGATGTGG -3´	ColE2-4 [fwd] sequencing
P17	5´- GTGGAATACGTGGATTGC -3´	ColE2-5 [fwd] sequencing
P18	5´- GGAGAAGCTATAAACCATG -3´	ColE2-6 [fwd] sequencing
P19	5´- TCTGCTCATGTTTGACAGCTT -3´	ColE2-7 [fwd] sequencing
P20	5´- CTCTGTTCGCATGGTCAG -3´	ColE2-8 [rev] sequencing
P21	5´- CACGTTCGATGTCGTTC -3´	ColE2-9 [rev] sequencing
P22	5´- GAATACATTCTCACACGCTC -3´	ColE2-10 [rev] sequencing
P23	5´- CGTTGTTGTTGCCTGTG -3´	ColE2-11 [rev] sequencing
P24	5´- TCATCCGCCAAAACAGCC -3´	ColE2-12 [rev] sequencing
P25	5´- ATTAAGTCGACGAAGATCCTTTGATCTTTTC -3´	pMO3_noORI SalI [rev] Cloning pMO3 vector without ORI
P26	5´- ATTAAGCATGCAACGCCAGCAACGC -3´	pMO3_noORI SphI[fwd] Cloning pMO3 vector without ORI
P27	5´- ATTAAGTCGACTTGAGATCGTTTTGG -3´	p15A ORI SalI [rev] cloning
P28	5´- ATTAAGCATGCTTTCCATAGGCTCCG -3´	p15A ORI SphI [fwd] cloning

Table S4. Sequences of genetic elements. The first three rows depict sequence changes in the LexA binding site (two overlapping LexA binding SOS boxes) on the pMO3 reporter plasmid, leading to altered LexA binding (pMO4, pMO5). The following three rows show the changes made in the CsrA binding site within the second mRNA loop (which also includes the ribosome binding site of the *cel* gene and the GGA motif recognized by CsrA) that potentiate (pMO6) or weaken (pMO7) CsrA binding (**Fig 2d**). The following three rows list the sequences of RNA oligos used for CsrA binding studies (**Methods, Fig 2d**) and include the alterations in the CsrA binding site mentioned above. The last three rows give the sequences of the 89-bp ssDNA oligos used to study binding of CsrA to ssDNA by gel shift analysis (**Methods**). Bases highlighted in *green* correspond to sequence changes. Bases shown in boldface highlight the GGA motif required for CsrA binding as present within the second mRNA (plasmid), RNA oligo or ssDNA oligo loop. We confirmed the appropriate formation of secondary structures of these oligos using Mfold(10) (**Methods**).

Name	Sequence	Description
pMO3	5‘-TTGATCTGTACATAAAACCAGTGGTTTTATGTACAGTATTAA-3‘	LexA binding site
pMO4	5‘-TTGTACTGTACATAAAACCAGTGGTTTTATGTACAGTATTAA-3‘	LexA binding site
pMO5	5‘-TTGATCCCTACATAAAACCAGTGGTTTTATGTACAGTATTAA-3‘	LexA binding site
pMO3	5' - CACAACAA**GGA**GTCGTTATG - 3'	CsrA binding site second loop
pMO6	5' - CACAACAA**GGA**TGTGTTATG - 3'	CsrA binding site second loop
pMO7	5' - CACATTAA**GGA**GTCGTTATG - 3'	CsrA binding site second loop
RNA Oligo equivalent to sequence of pMO3	5‘-Cy5- AUUUAAACAGGGCUGAAAUAUGAAUGCCGGUUGUUUAU **GGA**UGAAUGGCUGGCAUUCUUUCACAACAA**GGA**GUCGUUAUGA AAAAAUA -3‘	RNA Oligo with both CsrA binding sites (GGA)
RNA Oligo equivalent to sequence of pMO6	5‘-Cy5- AUUUAAACAGGGCUGAAAUAUGAAUGCCGGUUGUUUAU **GGA**UGAAUGGCUGGCAUUCUUUCACAACAA**GGA**UGUGUUAUGA AAAAAUA -3‘	RNA Oligo with both CsrA binding sites (GGA)
RNA Oligo equivalent to sequence of pMO7	5‘-Cy5- AUUUAAACAGGGCUGAAAUAUGAAUGCCGGUUGUUUAU **GGA**UGAAUGGCUGGCAUUCUUUCACAUUAA**GGA**GUCGUUAUG AAAAAAUA -3‘	RNA Oligo with both CsrA binding sites (GGA)
ssDNA Oligo equivalent to sequence of pMO3	5‘-Cy5- ATTTAAACAGGGCTGAAATATGAATGCCGGTTGTTTAT **GGA**TGAATGGCTGGCATTCTTTCACAACAA**GGA**GTCGTTATG AAAAAAATA - 3‘	ssDNA Oligo with both CsrA binding sites (GGA)
ssDNA Oligo equivalent to sequence of pMO6	5‘-Cy5-ATTTAAACAGGGCTGAAATATGAATGCCGGTTGTTTAT **GGA**TGAATGGCTGGCATTCTTTCACAACAA**GGA**TGTGTTATG AAAAAATA -3‘	ssDNA Oligo with both CsrA binding sites (GGA)
ssDNA Oligo equivalent to sequence of pMO7	5‘-Cy5- ATTTAAACAGGGCTGAAATATGAATGCCGGTTGTTTAT GGATGAATGGCTGGCATTCTTTCACATTAA**GGA**GTCGTTATG AAAAAATA -3‘	ssDNA Oligo with both CsrA binding sites (GGA)

Supplementary Figures

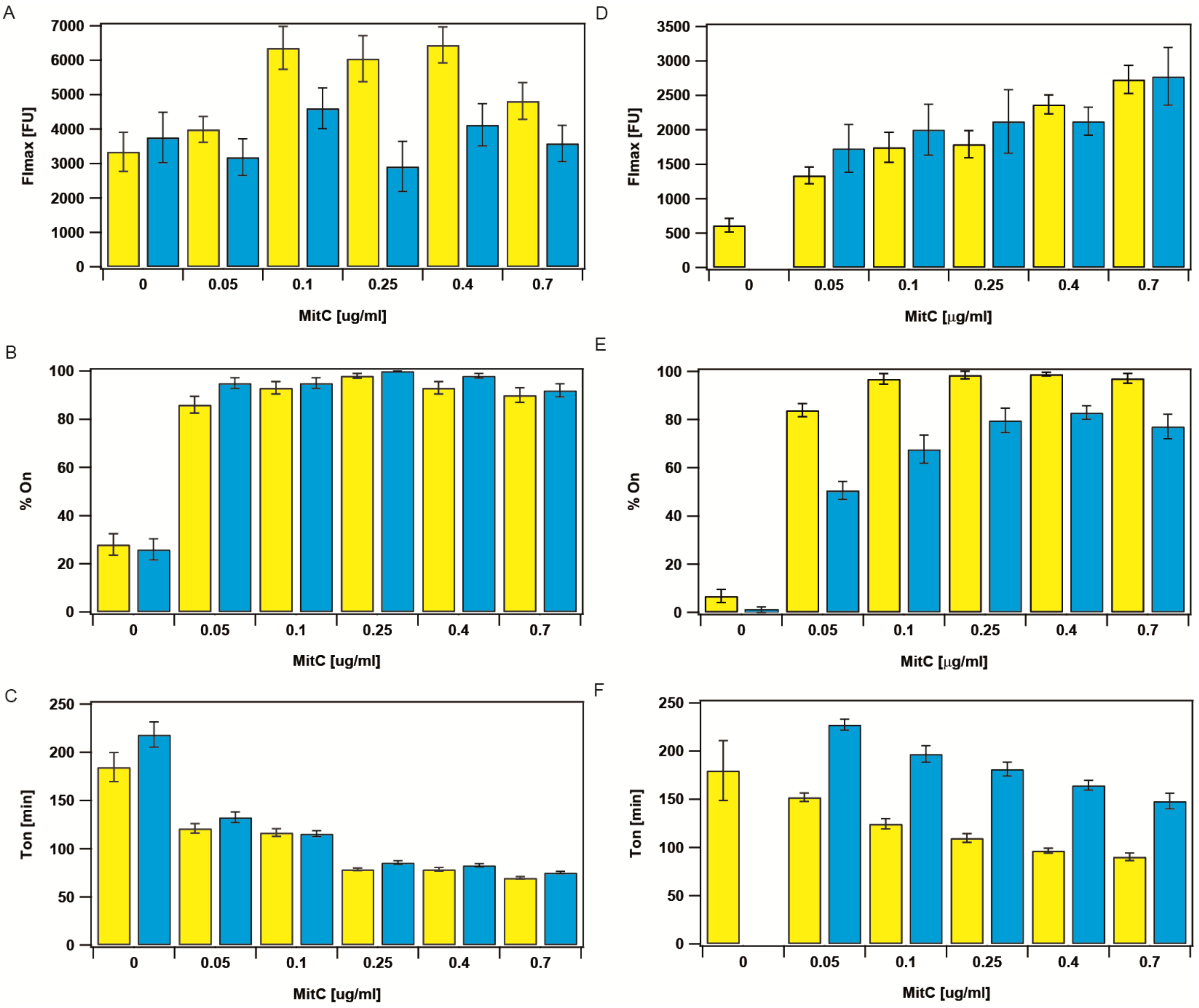

Fig S1: Dependence of the maximal fluorescence intensity, cumulative fraction and time-point of expression start of cells expressing the ColicinE2 operon in the C_{REP1} and S_{REP1} strains on the MitC concentration. Yellow: *cea* gene expression (colicin production), blue: *cel* gene expression (colicin release). Data shown here represent average values obtained from single-cell time-lapse microscopy experiments. A-C) C_{REP1}, D-F) S_{REP1}, A,D) Maximal fluorescence intensity of the cells that express the ColicinE2 operon. B,E) Cumulative fraction of cells expressing the ColicinE2 operon. C,F) Onset (t_{ON}) of *cea* and *cel* gene expression.

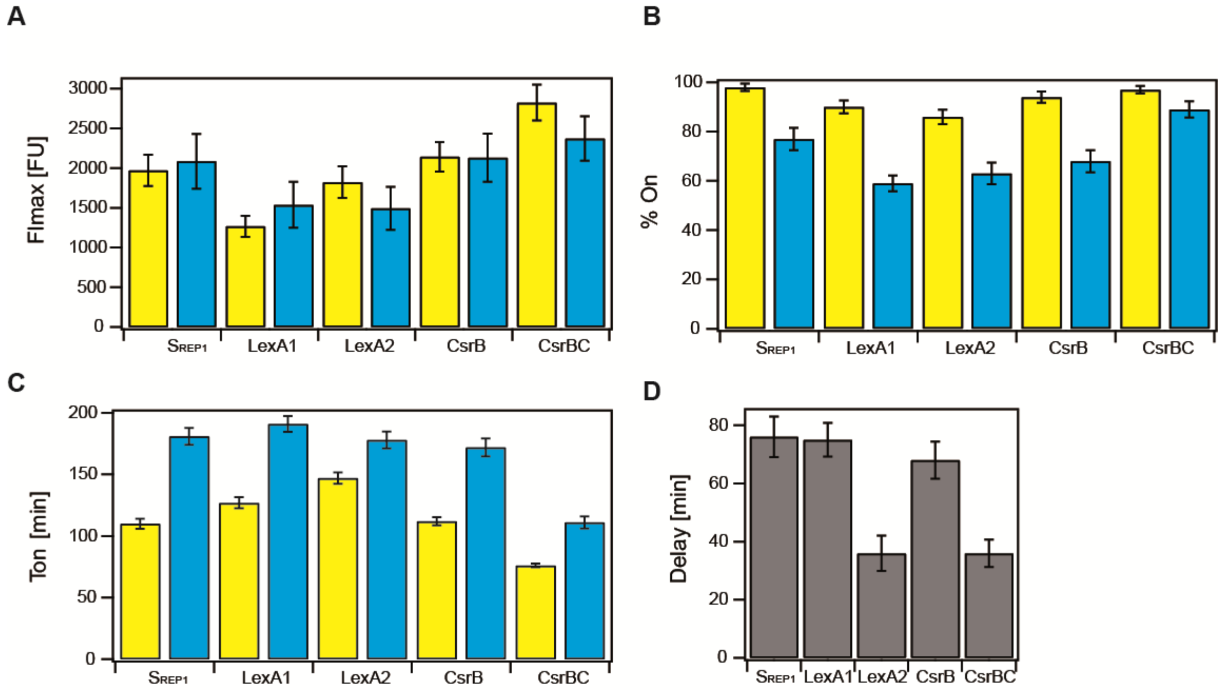

Fig S2: Impact of alteration of the LexA binding site or absence of sRNAs on ColicinE2 expression in S_{REP1} cells. A-C) Yellow: *cea* gene expression (colicin production), blue: *cel* gene expression (colicin release). **A)** Mean maximal expression of the colicin operon (relative to the S_{REP1} strain) in mutant reporter strains bearing altered LexA binding sites (LexA1 and LexA2, **SI, Methods**), or lacking the sRNA CsrB (CsrB), or missing both sRNAs CsrB and CsrC (CsrCB). **B)** Cumulative fraction of cells expressing the colicin operon. **C)** T_{ON} times for *cea* and *cel* gene expression. **D)** Delay between *cea* and *cel* gene expression.

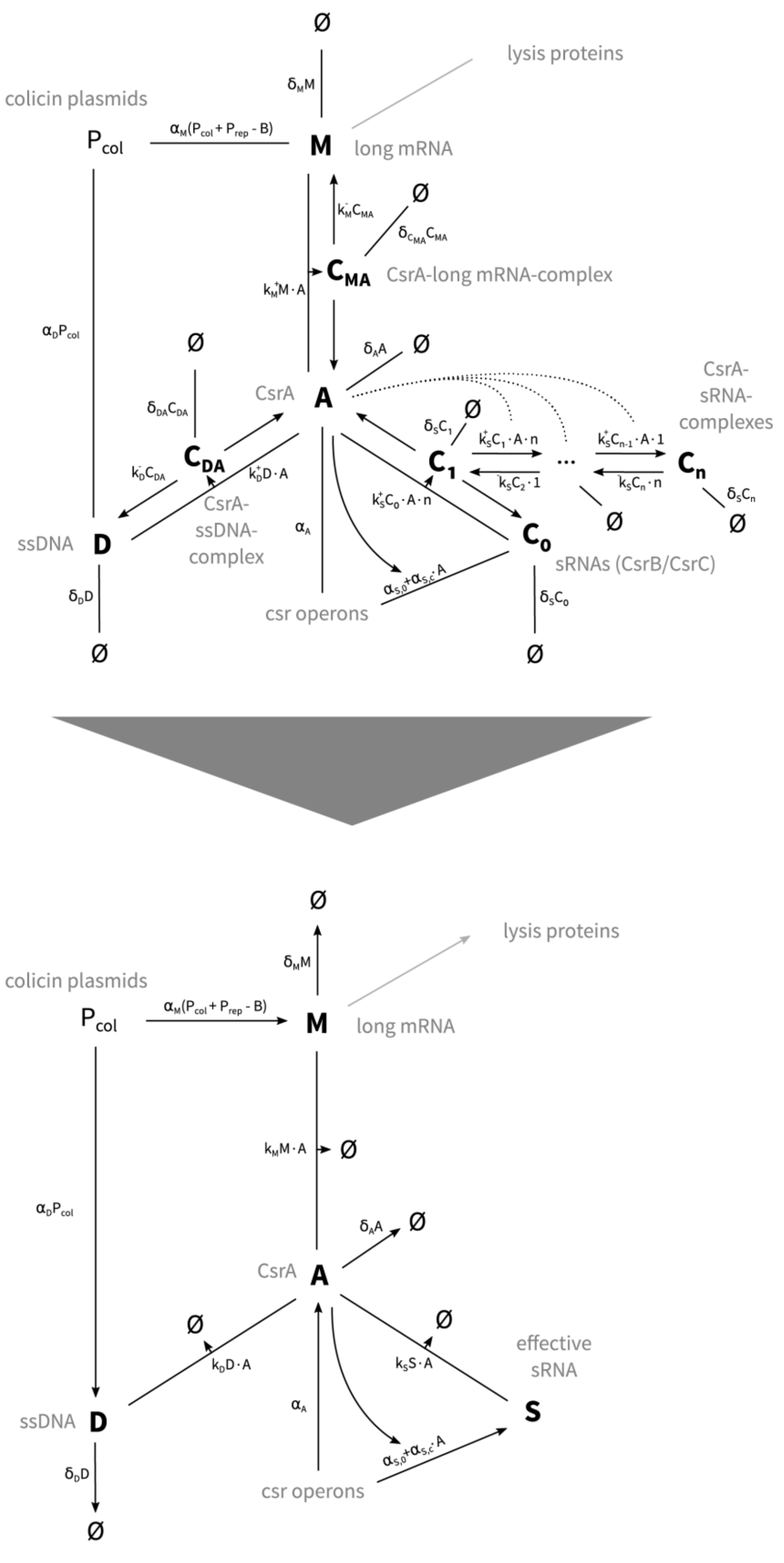

Fig S3: Biochemical network involved in the post-transcriptional regulation of ColicinE2. The top part shows the complete network, involving all interactions and components considered in this work. This complex description of the network can be reduced to the set of effective interactions shown in the lower panel. The derivation of these effective descriptions is given in section 2 of the theory part of the **SI.**

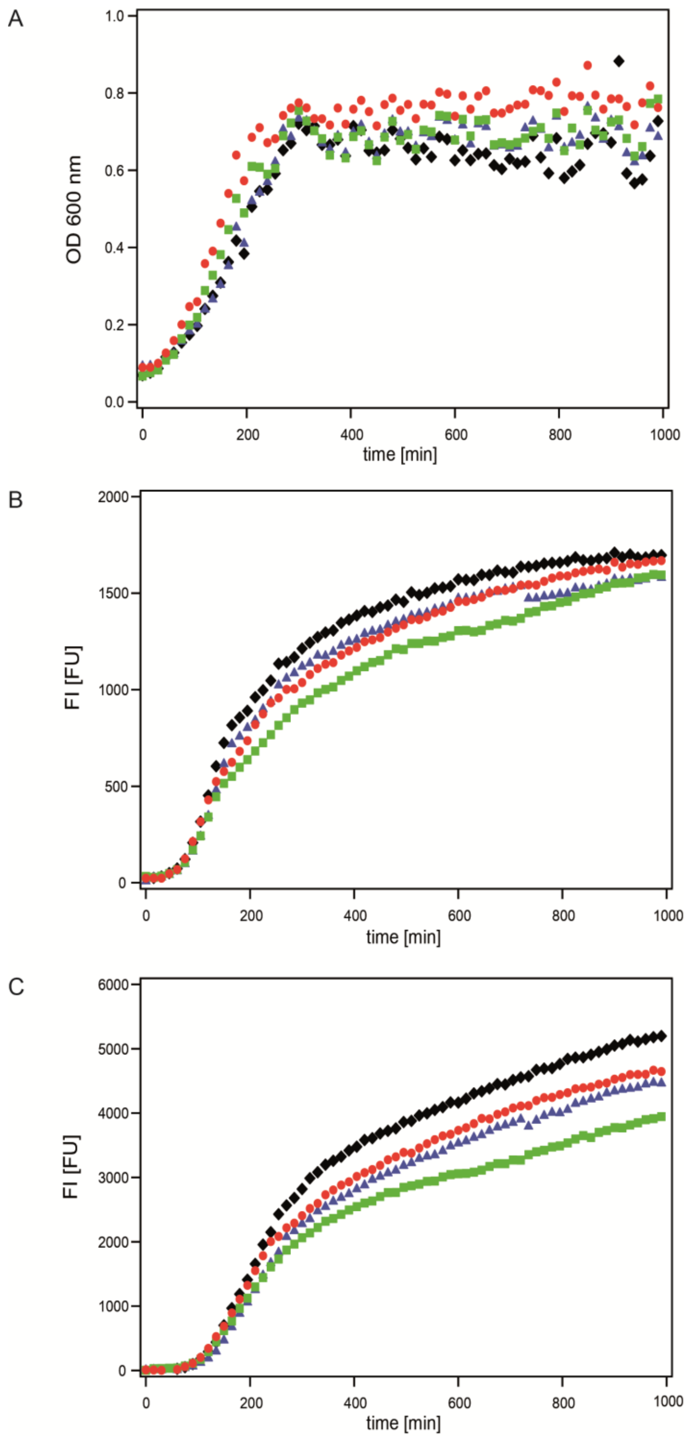

Fig S4: Effect of sRNA knock-out on Colicin E2 expression in long-term experiments (0.25 µg/ml MitC). To determine the importance of sRNA regulation for ColicinE2 expression over a longer period, plate-reader experiments were performed as described in **Methods**. **A)** Absorbance, **B)** Fluorescence intensity of cells expressing *cea* (YFP, colicin production), **C)** Fluorescence intensity of cells expressing *cel* (CFP, colicin release). Red: S_{REP1} strain, Blue: CsrC single sRNA knock-out, Green: CsrB single sRNA knock-out, Black: CsrB/C double sRNA knock-out.

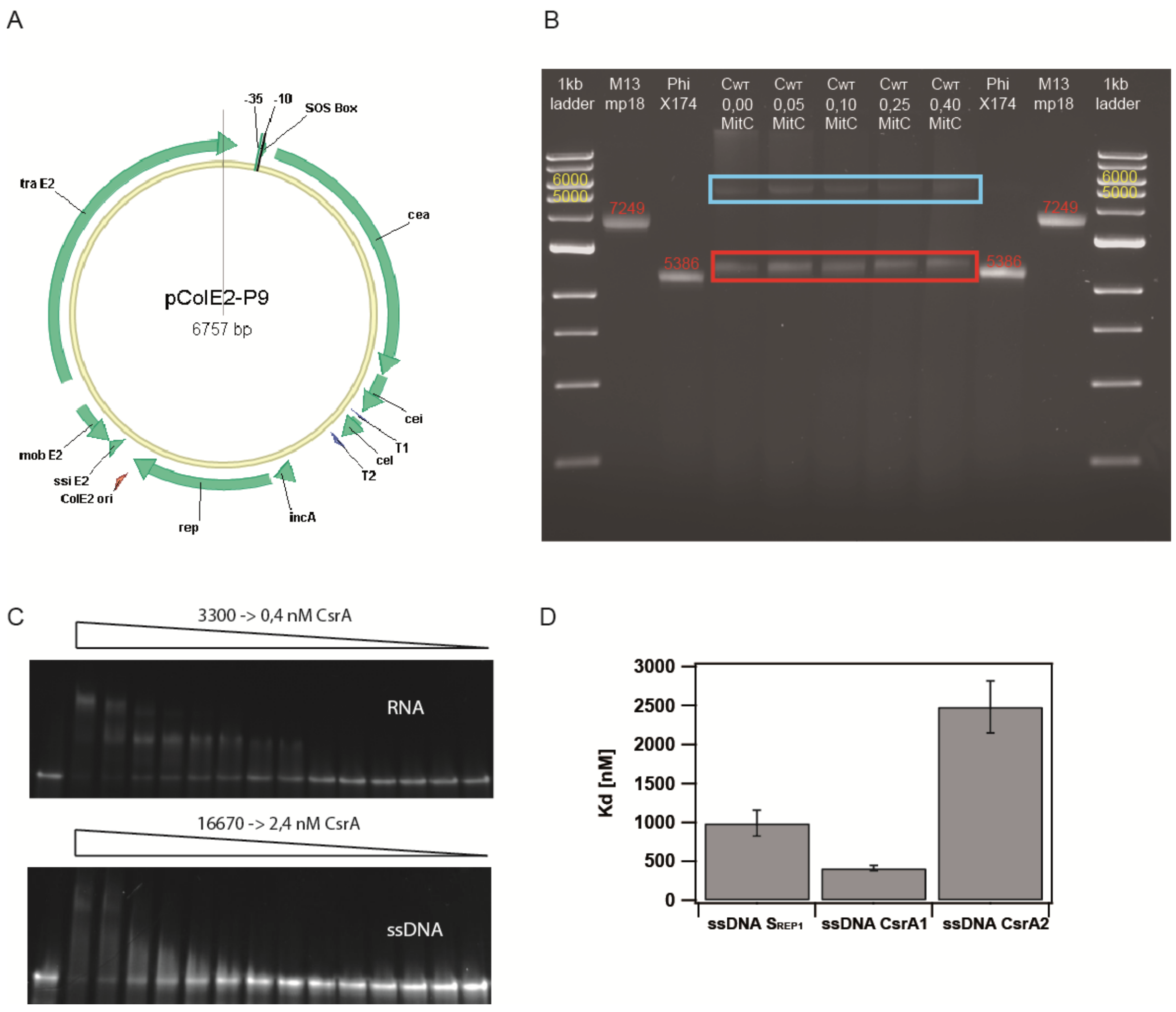

Fig S5: ssDNA accumulation in bacteria carrying pColE2-P9 and binding of CsrA to ssDNA. A) Map of pColE2-P9. The plasmid map was created using Vector NTI Expression Version 1.6.0. The plasmid sequence can be accessed via Genbank accession number KY348421. **B)** Accumulation of ssDNA in C_{WT} is independent of the presence of MitC. Agarose gel of plasmid and ssDNAs extracted from $C_{WT.}$ Lanes 1-3 and 9-11 were loaded with the indicated markers: 1-kb ladder, 7249-bp ssDNA ring (M13mp18), and 5386-bp ssDNA ring (PhiX174). Lane 4-8: uncleaved C_{WT} DNA showing the 6800-bp pColE2-P9 dsDNA (blue) and ssDNA (red) at different concentrations of the SOS inducing agent MitC. The staining substance ETBR binds optimal to dsDNA and only to ssDNA if secondary structures (ds part of ssDNA) are present. Hence, binding of ETBR to ssDNA is much lower than to dsDNA. Consequently, the brighter ssDNA bands reflect the high amount of ssDNA accumulated in the bacterial cells. **C**) Gel-shift analysis of CsrA binding to RNA or ssDNA oligos equivalent to the RNA corresponding to pMO3 (**Methods**, **SI**), verifying binding of 1 or 2 CsrA molecules to single RNA and ssDNA oligos (first and second shift, respectively). In the first lane for comparison no CsrA is added. RNA or ssDNA was applied at 5 nM. **D**) Dissociation constants (K_d) for the binding of CsrA to various ssDNA oligos (**SI**). CsrA1 = stronger CsrA binding, CsrA2 = weaker CsrA binding.

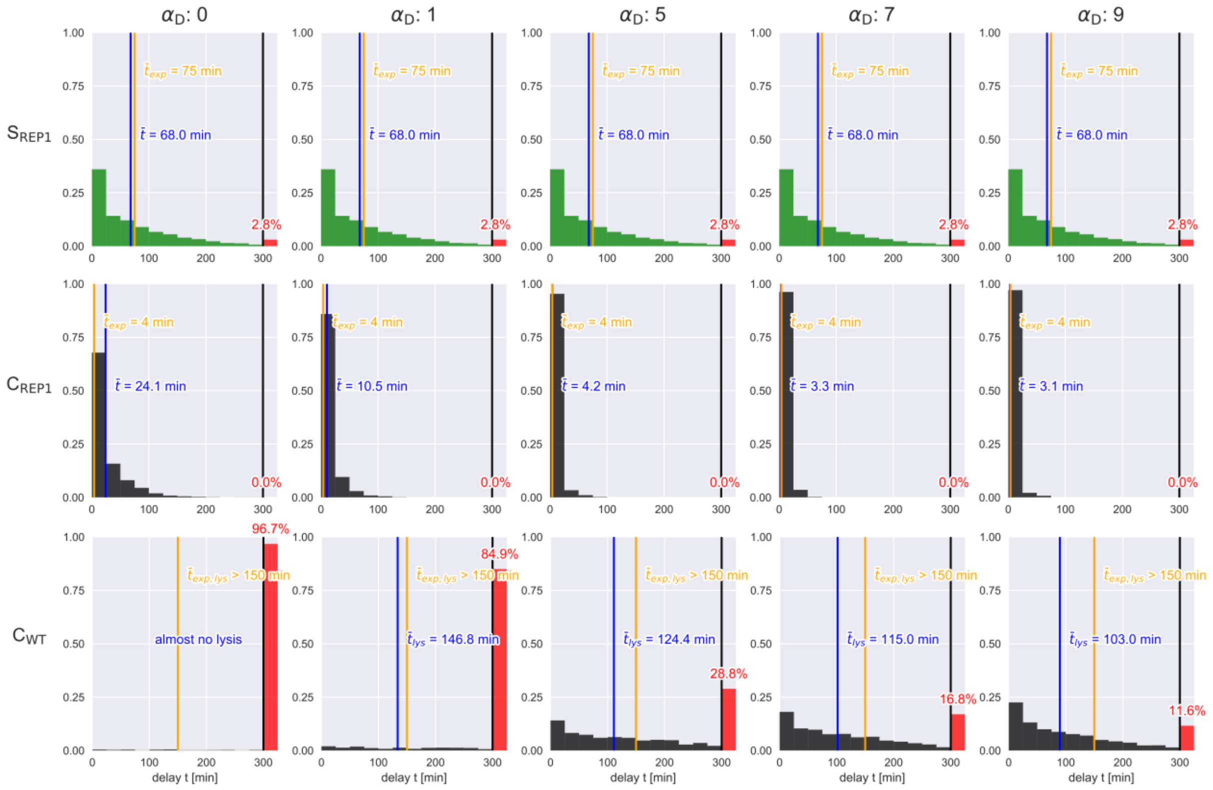

Fig S6: *cea-cel* delay-time distributions and average *cea-cel* delay-times for different ssDNA production rates and strains. The S_{REP1} strain does not produce ssDNA, and is plotted (in green) only for the purpose of direct comparison with the C_{REP1} strain. If no ssDNA is produced ($\alpha_D = 0$), we find that the C_{REP1} strain shows a broader *cea-cel* delay-time distribution, compared to the cases with ssDNA production. The wild-type strain C_{WT} does not lyse at all during the SOS signal for $\alpha_D = 0$. If we increase the ssDNA production rate, we find the experimentally observed behaviour that the C_{REP1} strain shows very short *cea-cel* delays. In the wild-type strain, a certain threshold rate of ssDNA production is required to induce a significant level of lysis, emphasizing the importance of ssDNA for toxin release. Ensemble size: 2000 realisations.

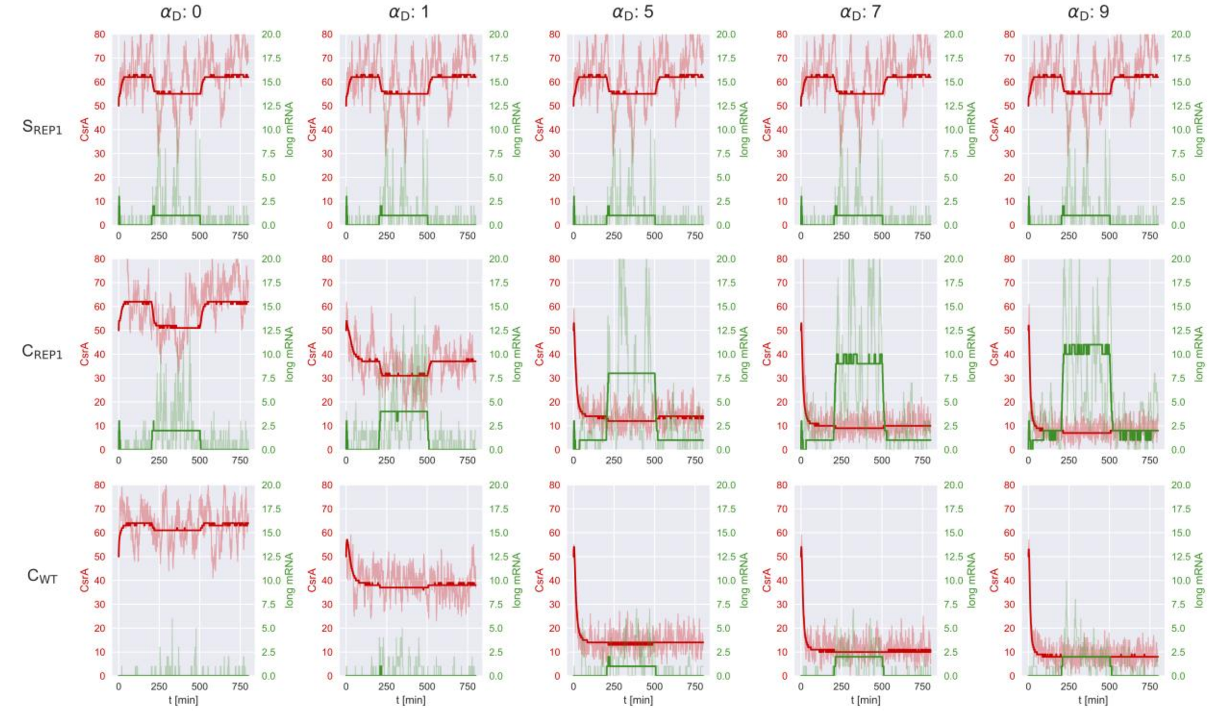

Fig S7: Average of the time evolution of the CsrA and long mRNA abundance for different ssDNA production rates and strains. Between t=200 and t=500, the system is subject to an SOS signal. In all cases, the SOS signal initiates a decrease in CsrA abundance from a previously stable level. This level is determined by the production, binding, and degradation rates of CsrA and its complex partners. As higher CsrA levels take longer and are also less likely to decrease to zero, they also directly affect the duration of the average *cea-cel* delay-time. The three plots for $\alpha_D = 0$ also show a single trajectory of long mRNA and CsrA in light green and light red, respectively. Ensemble size: 2000 realisations.

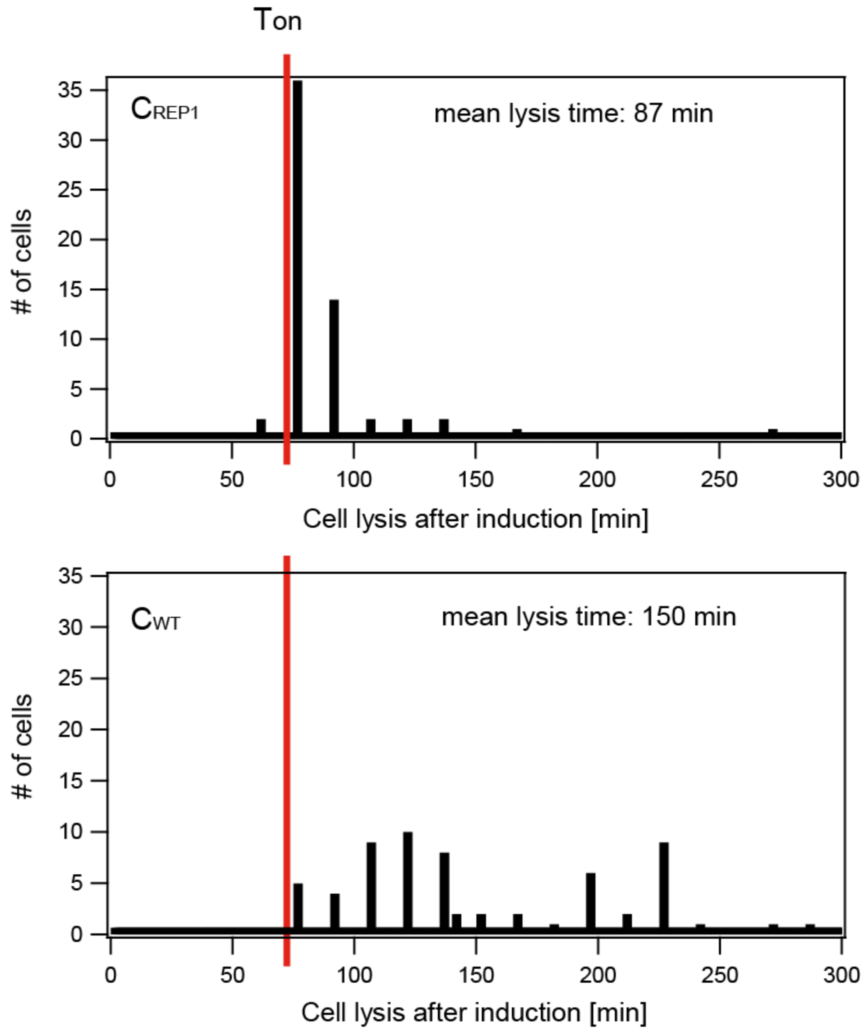

Fig S8: Cell lysis after induction with 0.25 µg/ml MitC. C_{REP1} lyses on average 87 (± 3.9) min after induction with MitC, C_{WT} lyses considerably later at 150 (± 6.9) min. For C_{REP1}, mean cell lysis nearly coincides with the T_{ONcea} at 69.71 ± 0.77 min(7) (red line). This interval between T_{ONcea} constitutes the delay between the SOS signal (MitC induction) and the start of *cea* gene expression. In our theoretical model, this initial delay is very short, as the system here switches directly from the pre-SOS signal 'OFF' state into the post-SOS signal 'ON' state. Hence, the red line also depicts the time-point of this switch (0 min in **Fig 4**).

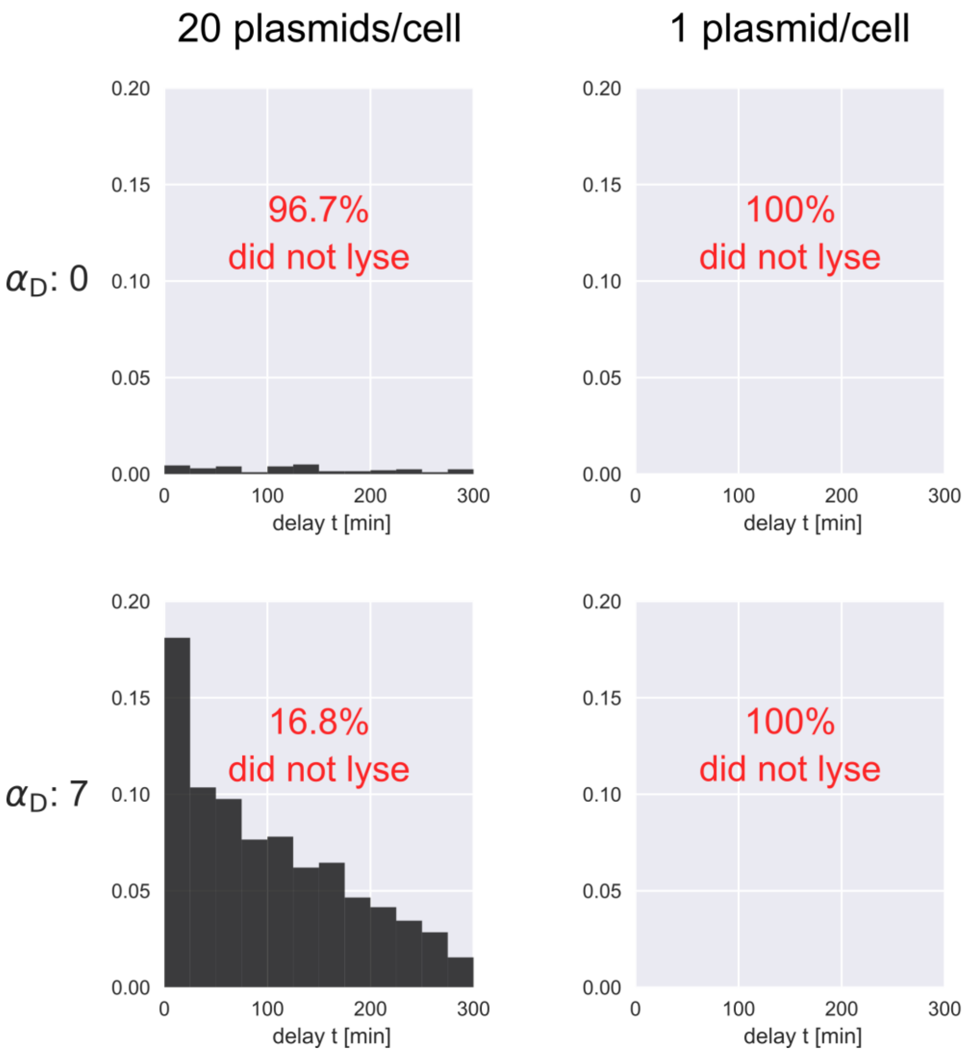

Fig S9: Importance of ssDNA production from the toxin producing plasmid (pColE2-P9) for toxin release in the wild-type strain C_{WT}. Left: Simulations for ColicinE2 expression from the pColE2-P9 plasmid with a plasmid copy number of ~20 in the absence (α_D = 0) and presence (α_D = 7) of ssDNA. Right: Simulations for ColicinE2 expression from the pColE2-P9 plasmid with a plasmid copy number of only 1 in the absence (α_D = 0) and presence (α_D = 7) of ssDNA. Here, the simulation lacking ssDNA production would resemble toxin production from a chromosomally encoded ColicinE2 operon. Ensemble size: 2000 realisations.

Supplementary References (experimental)

1. Morales M, Attai H, Troy K, Bermudes D. Accumulation of single-stranded DNA in Escherichia coli carrying the colicin plasmid pColE3-CA38. Plasmid. 2015;77:7-16. Epub 2014/12/03.
2. Khan SA. Plasmid rolling-circle replication: highlights of two decades of research. Plasmid. 2005;53(2):126-36. Epub 2005/03/02.
3. Yagura M, Nishio SY, Kurozumi H, Wang CF, Itoh T. Anatomy of the replication origin of plasmid ColE2-P9. Journal of bacteriology. 2006;188(3):999-1010. Epub 2006/01/24.
4. Aoki K, Shinohara M, Itoh T. Distinct functions of the two specificity determinants in replication initiation of plasmids ColE2-P9 and ColE3-CA38. Journal of bacteriology. 2007;189(6):2392-400. Epub 2007/01/24.
5. del Solar G, Kramer G, Ballester S, Espinosa M. Replication of the promiscuous plasmid pLS1: a region encompassing the minus origin of replication is associated with stable plasmid inheritance. Mol Gen Genet. 1993;241(1-2):97-105. Epub 1993/10/01.
6. Sugiyama T, Itoh T. Control of ColE2 DNA replication: in vitro binding of the antisense RNA to the Rep mRNA. Nucleic acids research. 1993;21(25):5972-7. Epub 1993/12/25.
7. Mader A, von Bronk B, Ewald B, Kesel S, Schnetz K, Frey E, et al. Amount of colicin release in Escherichia coli is regulated by lysis gene expression of the colicin E2 operon. PLoS One. 2015;10(3):e0119124. Epub 2015/03/10.
8. del Solar G, Giraldo R, Ruiz-Echevarria MJ, Espinosa M, Diaz-Orejas R. Replication and control of circular bacterial plasmids. Microbiol Mol Biol Rev. 1998;62(2):434-64. Epub 1998/06/10.
9. Kerr B, Riley MA, Feldman MW, Bohannan BJ. Local dispersal promotes biodiversity in a real-life game of rock-paper-scissors. Nature. 2002;418(6894):171-4. Epub 2002/07/12.
10. Zuker M. Mfold web server for nucleic acid folding and hybridization prediction. Nucleic acids research. 2003;31(13):3406-15. Epub 2003/06/26.

Supplementary Information (Theory): Theoretical Model for ColicinE2 Expression Including the Additional CsrA Sequestering Element ssDNA

1 The biological system

The biological phenomenon we wish to describe by means of a quantitative theoretical model is the regulation of ColicinE2 release in *E. coli*. ColicinE2 is a bacterial toxin encoded by the gene *cea*, which is part of the ColicinE2 operon located on a plasmid. This operon also contains genes for an immunity protein (*cei* gene) and a lysis protein (*cel* gene). The lysis protein is part of the operon as cell lysis is the only way to release the toxin into the environment. Since lysis also means the death of the cell, the release of ColicinE2 is highly regulated. Previous studies have revealed the regulatory components controlling ColicinE2 production and release on both the transcriptional and post-transcriptional levels:

- The *transcription* of the operon is regulated by the repressor *LexA*, which is part of the *E. coli* SOS response regulatory network [1, 2]. Stressful events, such as DNA damage activate a SOS response system [1], which stochastically triggers the transcription of the operon by degradation of *LexA*. Once transcription starts, two mRNA transcripts are produced: *short mRNA*, containing only the toxin and immunity protein, and *long mRNA*, which contains also the lysis protein [3].
- The *post-transcriptional* regulation (see Fig. 1 and also Fig. S3) acts on the long mRNA only. To our knowledge, its only regulator is the protein *CsrA*, which binds to the Shine-Dalgarno sequence that

is located on the long mRNA between the sequences coding for the immunity and lysis proteins [3]. When a CsrA protein binds to long mRNA and thus forms a complex with it, the gene for the lysis protein can no longer be translated, and thus the cell does not lyse [3]. By preventing lysis protein expression, CsrA regulates the release of ColicinE2. The abundance of CsrA itself is known to be regulated by the two CsrA-sequestering short RNAs (sRNAs) *CsrB* and *CsrC*, which both have several CsrA binding sites [4, 5]. Our current study suggests that, in addition, rings of single-stranded DNA (ssDNA) also sequester CsrA, and therefore represent a novel CsrA regulator. This ssDNA is created as an intermediate during the rolling circle replication of the ColicinE2 plasmids.

A particular example for the importance of these regulatory interactions is the delay between production and release of ColicinE2, which has recently been studied experimentally [6]: The translation of lysis proteins from long mRNA (and therefore lysis itself) can only start if there are free long mRNAs, that is, long mRNAs that are not bound to CsrA. From what is known about CsrA interactions, we assume in our biochemical model that a CsrA molecule can no longer regulate long mRNA when it is either sequestered, or degraded. Previous studies [7] show that CsrA is highly abundant during growth phase, mainly in form of CsrA complexes. When an SOS response is triggered, however, the production of long mRNA increases such that free CsrA abundance decreases, and eventually lysis proteins are translated from free long mRNA. This process does not happen instantaneously: Due to stochasticity in the SOS response system and the time it takes to produce, bind or degrade the regulatory components involved, we find a delay between the expression of the unregulated *cea* gene (part of the short mRNA) and the CsrA-regulated *cel* gene (part of the long mRNA). This delay is presumably not just a byproduct of regulation, but has also a biological function: It gives the cell time to accumulate ColicinE2, and thus allows for higher toxin concentrations upon the release. Moreover, it presumably also acts as a safety buffer, which prevents premature cell lysis, for instance due to fluctuations in the system [6].

In contrast to the abundances of the regulatory components, the *cea-cel*-delay is a quantity that can readily be measured experimentally by *reporter plasmids* inserted in the *E. coli* cells. These reporter plasmids carry the ColicinE2 operon, but the toxin (*cea*) and lysis (*cel*) gene are replaced by two different fluorescence protein genes (CFP and YFP). Since only these two

strain	number of reporter plasmids	number of pColE2P9 plasmids	ssDNA
S_{REP1}	≈ 55	–	–
S_{REP2}	≈ 13	–	–
C_{REP1}	≈ 55	≈ 20	accumulates
C_{REP2}	≈ 13	≈ 20	accumulates
C_{WT}	–	≈ 20	accumulates

Table S5: The five different strains and the abundance of the genetic elements that differentiate them [8].

genes are replaced, the reporter plasmids have the same promoter as the ColicinE2 plasmid, and the long mRNA transcript of the reporter plasmid has the same CsrA binding site as the original long mRNA. Consequently, a reporter plasmid behaves like the ColicinE2 plasmid, but produces two types of fluorescence proteins instead of toxin and lysis proteins. Therefore, upon introducing reporter plasmids to an *E. coli* cell, one can measure the time points of production of the corresponding fluorescence proteins; these measured time points then coincide with the time points of toxin and lysis protein production. In this study, we use two reporter plasmid types, which differ by their mean abundance in the cell: type 1 (pMO3) accumulates to about 55 plasmids per cell, whereas type 2 (pMO8) only to about 13 plasmids per cell.

With three different plasmid types, the original and the two reporter plasmids, we can construct five different strains (see also Table S5): First, the wild-type strain, C_{WT}, which carries only ColicinE2 plasmids. Inserting reporter plasmids to this strain creates, depending on the reporter plasmid type inserted, either a strain called C_{REP1} or a strain called C_{REP2}. Completely replacing the ColE2 plasmid with one of the reporter plasmid types creates another two strains, referred to as S_{REP1} and S_{REP2}. Our experiments show that the plasmid types also differ in the production of ssDNA: cells carrying the ColicinE2 plasmid do accumulate ssDNA (see Fig. 3), while this is not the case for the S_{REP1} and S_{REP2} strain cells, which only contain reporter plasmids (see Fig. 3 and Supplementary Information).

As discussed in detail in section 2, we will use mathematical modelling to infer the delay of the wild-type strain from the delay measured in the other strains containing the reporter plasmid. Before doing so, we recapitulate the main experimental findings on the delay-times in the bacterial strains containing the reporter plasmid.

In our experiments (see main text) we found that the C_{REP1} strain shows

no significant delay between *cea* and *cel* gene expression, whereas the S_{REP1} strain has a significant mean delay of 75 minutes. Moreover, we observed that the S_{REP1} strain has a broad delay-time distribution around this mean value. This raised the question as to the source of this difference. The C_{REP1} and S_{REP1} strains differ only in their plasmid composition and are both genetically identical (see Table S5). From this we conclude that the presence of ColicinE2 plasmids in the C_{REP1} strain introduces further regulatory elements (compared to the reporter plasmids), which are responsible for the shorter *cea-cel* delay times compared to the S_{REP1} strain. The reporter and the ColicinE2 plasmids contain the same regulatory sequences (see **Methods**), which means that the additional regulatory elements cannot be different mRNA transcripts specifically produced by the ColicinE2 plasmid. From what is known about the two plasmids and the regulatory network of ColicinE2 (see above), two mechanisms could in principle account for the shorter delay times in C_{REP1}, which are: First, additional production of CsrA sequestering long mRNA due to the larger plasmid copy number, and second, the accumulation of ssDNA which, as our study shows, can also sequester CsrA.

2 Mathematical model of the ColicinE2 release

In the following, we develop a mathematical model that enables us to investigate the regulation of ColicinE2 release in all five strains (C_{REP1}, C_{REP2}, C_{WT}, S_{REP1}, and S_{REP2}). The model accounts for all necessary regulatory components, including the ssDNA and the different plasmid compositions. We validate this model by reproducing the experimentally observed delay time distributions for the S_{REP1} strain. Variation of ssDNA production in the model then allows us to quantify the impact of ssDNA production and plasmid copy number on the *cea-cel* delay. Moreover, the model enables us to infer the behaviour of the C_{WT} strain, for which the *cea-cel* delay cannot be directly measured experimentally. The inferred behaviour can be validated by comparison with experimentally measured lysis times, see Fig. S8.

Several experimental studies defined and probed the regulatory networks and components involved in *E. coli* SOS responses, as well as ColicinE2 production and release [1, 3, 6, 8]. Starting from these experimental results, the regulatory interactions have also been studied using mathematical models [2, 9–11]: For the transcriptional regulation network of the *E. coli* SOS response system, a stochastic model has been presented, which is able

to reproduce the distribution of stochastic SOS activity peaks [2]. For the post-transcriptional regulation of ColicinE2 release, we recently introduced a hierarchical three-component model [9] involving long mRNA, CsrA, and an effective sRNA. This model was also combined with the stochastic SOS signal model from Ref. [2] to emulate the response of ColicinE2-producing bacteria to external stress. With this combined approach, the model shows that sRNA reduces internal fluctuations and helps controlling the level of CsrA. Moreover, the model predicts stochastically distributed delays between SOS signal and lysis, which is also seen in experiments with the S_{REP1} strain.

In this section, we extend our previous model [9], taking into account the new experimental findings presented in the main text. In particular, we incorporate the additional regulator ssDNA as well as the different plasmid copy numbers and types. For this step, it is important to know the derivation of the previous, three-component model, which is why we outline the derivation of the previous model as we develop our new model from scratch. For a detailed derivation of the three-component model, we refer the reader to Ref. [9].

2.1 Regulatory network

Our goal is to design a stochastic model that enables us to investigate the dynamics of the regulatory networks involved the SOS response and the ensuing synthesis and release of ColicinE2. To this end, we first formulate the interactions of the regulatory components as a set of (deterministic) differential equations, that is, as a mass-action model. This approach disregards any spatial effects and considers the system as *well mixed*.

Extending our previous study [9], we build a mass-action model for the SOS response, and the regulatory network for ColicinE2 production and release from the following assumptions and properties of the components (see also Fig. S3):

- The abundances of long mRNA, CsrA and effective single-binding-site sRNA (see below) are denoted by M, A and S, respectively. These abundances give the number of *free* components, that is, the number of long mRNA, CsrA and sRNA molecules that are not bound in a complex. Moreover, P_{COL} and P_{REP} denote the copy number of ColicinE2 and the reporter plasmids, respectively (there is no need to distinguish between the two reporter plasmid types for P_{REP} as they do not occur in the same cell at the same time).

- The response to external stress ("SOS response") is regulated by the LexA/RecA system [1], which we incorporate into our model using the differential equations given in Ref. [2]. This model accounts for the production, degradation and (un)binding of the proteins LexA (L) and RecA (R), the mRNAs they are translated from (M_l and M_r, respectively), as well as the number of repressed promoters controlling the transcription of these mRNAs (B_l and B_r, respectively). In this system, LexA acts as repressor: as long as a LexA protein is bound to the promoter region of the RecA or LexA operon, no mRNA is produced. The number of repressed RecA and LexA promoters increases if LexA binds to an unrepressed promoter, and decreases as it unbinds. Therefore, the differential equations for B_l and B_r contain two terms each: a production term proportional to the abundances of LexA and unrepressed promoters, and a degradation term proportional to the number of repressed promoters. In an *E. coli* cell, there is only one promoter for each LexA and RecA, which means that B_l and B_r can take either the values 0 or 1. The differential equations then read

$$\partial_t B_r = k_r^+(1 - B_r)L - k_r^- B_r, \tag{1}$$

$$\partial_t B_l = k_l^+(1 - B_l)L - k_l^- B_l, \tag{2}$$

where the $k^\pm$ denote the attachment and detachment rates of LexA to/from the promoter indicated by the subscript. From unrepressed promoters the respective mRNA is transcribed, and hence, the mRNA production depends linearly on the number of unrepressed promoters. Once produced, the mRNA can spontaneously degrade. Therefore, the differential equations for the mRNAs also contain two terms each, and read:

$$\partial_t M_r = \alpha_{M_r}(1 - B_r) - \delta_{M_r} M_r, \tag{3}$$

$$\partial_t M_l = \alpha_{M_l}(1 - B_l) - \delta_{M_l} M_l, \tag{4}$$

where α and δ give the per capita production and degradation rate of the component indicated by the subscript. These mRNAs are translated to RecA and LexA proteins, respectively. Hence, the production terms of the two proteins are proportional to the respective mRNA abundance. The number of proteins decreases by spontaneous degradation. As the abundance of RecA is only affected by these two processes, its differential equation reads:

$$\partial_t R = \alpha_R M_r - \delta_R R, \tag{5}$$

where α_R and δ_R denote the per capita production and degradation rate of RecA. Since LexA acts as regulator in the LexA/RecA-system, its abundance is also affected by the interactions with the promoters. Consequently, the terms from eqs. (1) and (2) appear in the differential equation for LexA, but with opposite sign. Moreover, in case of an SOS signal, RecA depletes LexA, motivating an additional degradation term bilinear in L and R, with the degradation constant c_p. Apart from these interactions with the LexA/RecA-system, LexA is also the repressor of the colicin operon. Therefore, the LexA/RecA SOS response system interacts with the regulatory system of ColicinE2 production and release via B, the number of repressed promoters of the ColicinE2 operon. Its differential equation contains two terms analogous to the LexA and RecA promoters:

$$\partial_t B = k_p^+(P_{COL} + P_{REP} - B)L - k_p^- B, \tag{6}$$

where the $k_p^\pm$ denote the attachment and detachment rates of LexA repressor to/from the ColicinE2 promoter. Unlike B_r and B_l, B can take values between 0 and $P_{COL} + P_{REP}$. The terms from eq. (6) apppear, again with opposite sign, also in the differential equation for LexA, which, altogether, reads

$$\begin{aligned} \partial_t L = {} & \alpha_L M_l - \delta_L L - k_l^+(1 - B_l)L + k_l^- B_l - k_r^+(1 - B_r)L \\ & + k_r^- B_r - k_p^+(P_{COL} + P_{REP} - B)L + k_p^- B - c_p RL, \end{aligned} \tag{7}$$

where α_L and δ_L give the per capita production and degradation rate of LexA. For a detailed discussion of these equations, we refer to Ref. [2]. Note that the SOS response system, eqs. (1)-(7), interacts with the ColicinE2 regulatory network only through the parameter B (see also next bullet point).

- The total production rate of long mRNA in the cell is proportional to the number of unrepressed ColicinE2 promoters in the cell. This number is given by the total number of plasmids in the cell, $P_{COL} + P_{REP}$, minus B, the number of promoters with the repressor LexA bound to it. Hence, the production rate of long mRNA reads

$$\alpha_M(P_{COL} + P_{REP} - B), \tag{8}$$

where α_M is the production rate per unrepressed promoter. Note that considering different plasmid types generalizes our earlier work presented in Ref. [9].

- CsrA is produced at a constant rate, α_A.
- The ColicinE2 system has two different regulatory sRNAs: CsrB and CsrC. Apart from having different numbers of CsrA binding sites and slightly different half-lifes, their mode of binding with CsrA is very similar. Hence, we assume that we can describe their regulatory impact by a single *effective sRNA* with corresponding effective parameters (see Ref. [9] for details). Using effective sRNAs in a mathematical model is indeed supported by experiments, which show that the knock-out of either CsrB or CsrC causes a compensating overproduction of the other sRNA (see the main text, and Ref. [5]). This compensation is a natural consequence of a positive regulatory effect of CsrA abundance to sRNA production (see bullet point below), and highlights the functional equivalence of CsrB and CsrC. In Ref. [9] we also showed that this effective sRNA, which contains $N \approx 10$ CsrA binding sites is equivalent to N *effective single-binding-site sRNAs*. This drastically reduces the mathematical complexity of the model.
- Several studies found that the production of CsrB and CsrC is indirectly regulated by the abundance of CsrA via the BarA/UvrY-system [3–5]. Since the details of this interaction are largely unknown, we model this positive regulation with an sRNA production rate that is a linear function of the CsrA abundance. In addition to this linear term, we also introduce a constant baseline production term, since studies show that sRNAs are also produced (at very low levels) in the absence of free CsrA [5]. Both production terms contain a factor N, as we consider effective single-binding-site sRNAs in our model (see the previous bullet point). The production term of the effective single-binding-site sRNAs thus reads

  $$\alpha_{S,0} N + \alpha_{S,c} A \cdot N,$$

  with the baseline production rate $\alpha_{S,0}$, the linear coupling coefficient $\alpha_{S,c}$, and the abundance of CsrA proteins A. Note that we did not consider the positive feedback of CsrA on sRNA production in Ref. [9].
- The degradation rates of long mRNA, CsrA and the effective sRNA are each proportional to their respective abundance, and read $\delta_M M$, $\delta_A A$ and $\delta_S S$, respectively.
- CsrA can bind to both long mRNA and the effective sRNA, and thus forms CsrA-long mRNA and CsrA-sRNA complexes (C_{MA} and C_{SA},

respectively). In line with previous studies [12, 13] and our three-component model [9], we assume that the formation and disassembly of these complexes is much faster than the other processes involved in post-transcriptional regulation. Therefore, we can employ adiabatic elimination, $\partial_t C_{MA} \equiv 0$ and $\partial_t C_{SA} \equiv 0$. In Ref. [9], we show that this enables us to combine the formation, disassembly and degradation of the complexes into effective binding parameters, k_M and k_S. As a consequence, we can solve for the complex abundances, C_{MA} and C_{SA}, and eliminate them from our set of differential equations (see Ref. [9] for details).

- The precise mechanism for the degradation of CsrA-sRNA and CsrA-long mRNA complexes is not known. Here, we assume that *CsrA dimers are always degraded once their complex partner is degraded* (in other words: CsrA cannot "survive" the degradation of its partner).

- CsrA is a main regulator in growing *E. coli* cells, which is known to bind to over 700 different targets [14, 15]. In the Supplementary Information of Ref. [9] we show how one can eliminate the many targets of CsrA to obtain a reduced system, which contains only the components that are changed by the processes the model focusses on (in this case: SOS-induced production and release of ColicinE2). In the mathematical model presented in this section, we reduce the system to three CsrA targets: long mRNA, sRNA, and (see below) ssDNA. In agreement with experiments [7], the production rate of the effective sRNA is large compared to the production of long mRNA and ssDNA, such that the vast majority of sequestered CsrA proteins is bound to sRNA.

- The short mRNA is not regulated by CsrA, and hence not part of the regulatory network. However, our experiments use the translation of short mRNA (specifically, the translation of the *cea* gene) as proxy for promoter activity in the S_{REP1}, S_{REP2}, C_{REP1}, and C_{REP2} strain. To enable the experimental validation of our model, we include the production of short mRNA in our model. Due to the lack of regulation, the corresponding differential equation is decoupled from M, A and S, and reads (with $\frac{\partial M_{short}}{\partial t} \equiv \partial_t M_{short}$)

$$\partial_t M_{short} = \alpha_{M_{short}} - \delta_{M_{short}} M_{short}, \qquad (9)$$

where $\alpha_{M_{short}}$ and $\delta_{M_{short}}$ are the rate constants for production and per-capita degradation, respectively.

The properties and assumptions of the SOS response and the ColicinE2 regulatory system we listed above have already been used (if not stated otherwise) in the combined model for SOS response and ColicinE2 regulation presented in Ref. [9].

ssDNA as regulatory component: In the main text, we show experimentally that single-stranded DNA (ssDNA) serves as a component of post-transcriptional regulation of ColicinE2 production and release. Since this is a novel and, so far, an undocumented role of ssDNA, we briefly discuss how it acts as a regulator for CsrA in an *E. coli* cell.

ssDNA is an intermediate in the rolling circle replication mechanism of the ColicinE2 plasmid: The plasmid consists of double-stranded DNA (dsDNA). The first step in its replication is the production of a ring-shaped ssDNA transcript. These transcripts are produced both in absence and presence of an SOS signal (Fig. S5), which means that ssDNA production is constant. It is assumed that once a ring of ssDNA is completed, it detaches from the plasmid and diffuses freely through the cell. During this time, it is converted to double-stranded DNA, which eventually results in a new plasmid. Between the detachment of the single-stranded ring and the formation of a new plasmid, the ssDNA acts as a regulator of CsrA: Since the ssDNA includes the coding sequences present in the long mRNA, CsrA can bind to the Shine-Dalgarno sequence of the *cel* gene located on the ssDNA, and thus forms an ssDNA-CsrA complex. This allows the ssDNA to regulate free CsrA levels by sequestration, similar to the CsrA regulation by sRNA.

In our mathematical model, we account for these properties of ssDNA as follows:

- The production rate of ssDNA is assumed to be proportional to the number of ColicinE2 plasmids, P_{COL}, as it is an intermediate product of the rolling circle replication mechanism of the ColicinE2 plasmid. It reads

$$\alpha_D \cdot P_{COL}, \tag{10}$$

  with the per plasmid production rate constant α_D.

- The degradation of ssDNA is proportional to the ssDNA abundance, D, and thus reads

$$\delta_D \cdot D, \tag{11}$$

  with the per capita degradation rate constant is δ_D.

- The ssDNA has two binding sites for a CsrA dimer (see **Methods** of the main text), and thus can form a complex, C_{DA}, with it. Complex formation occurs with rate k_D^+, and the complexes dissociate into ssDNA and a CsrA dimer with rate k_D^-. Apart from disassembly, we also include the possibility that a CsrA-ssDNA-complex can spontaneously degrade (meaning that both CsrA and ssDNA are degraded at the same time) by introducing the per capita rate δ_{DA}. The abundance of complexes is denoted by C_{DA}.

Taken together, we can now formulate a set of differential equations, which allows us to quantify these interactions. These interactions are also illustrated as a biochemical network in Fig. S3.

We begin with the differential equation for the time evolution of the long mRNA, M. From the properties collected above, we conclude that this equation must contain three terms: The first term describes the production of long mRNA, which is proportional to the number of unrepressed promoters. This number is calculated from the difference between the total plasmid copy number, $P_{COL} + P_{REP}$, and the number of repressed promoters, B. The abundance of long mRNA is reduced by a second and a third term: The second term describes the spontaneous degradation of long mRNA, and is proportional to its abundance, M. The third term is bi-linear (that is, it is proportional to A and M) and represents the effective coupled degradation of long mRNA in complexes with CsrA. This term combines the binding of CsrA to long mRNA, the dissociation of this complex, and its degradation in an effective binding parameter k_M. The three terms read:

$$\partial_t M = \alpha_M (P_{COL} + P_{REP} - B) - \delta_M M - k_M M \cdot A. \quad (12)$$

The derivation of the effective coupled degradation in the third term is described in detail in Ref. [9]; an analogous derivation for the ssDNA is given below. The B in the first term is determined by the LexA/RecA subsystem of the SOS response, in particular by eq. (6).

The differential equation for the time evolution of the effective single-binding-site sRNA, S, consists of terms very similar to that for long mRNA. Two terms account for spontaneous and effective coupled degradation, respectively, and are structurally the same as in eq. (12). This is due to the fact that the sRNAs regulate CsrA in the same way as CsrA regulates the long mRNA, by forming complexes. The production term is, however, different, and contains two parts: The first part, $\alpha_{S,0}$, describes a constant baseline production, which ensures the production of sRNAs in the absence of CsrA. The second part depends linearly on the abundance of free CsrA,

and thus accounts for the positive regulatory function of CsrA for the sRNAs. Taken together, these four terms give the differential equation for S:

$$\partial_t S = \alpha_{S,0} N + \alpha_{S,c} N \cdot A - \delta_S S - k_S S \cdot A. \tag{13}$$

Having described the two partners of CsrA, we now turn to the differential equation for CsrA itself. Again, this equation has a very similar structure to eqs. (12) and (13): An, in this case constant, production term, as well as a term for spontaneous degradation. Here, however, we have more than one coupled degradation term, since CsrA can bind to more than one component: long mRNA (M), sRNAs (S), and ssDNA (D). The effective coupled degradation terms for long mRNA and sRNA are exactly the same as in eqs. (12) and (13), respectively. This reflects the fact that the formation of a long-mRNA/CsrA- or sRNA/CsrA-complex has for both complex partners the same consequence, that is, it reduces the abundance of free CsrA by 1. The coupled degradation part is also responsible for the hierarchical regulation, which we discussed in [9]: The actual regulation target, long mRNA (M), exclusively binds to CsrA; the sRNAs affects the free long mRNA level only indirectly by sequestering the CsrA and thus "regulating the regulator". Moreover, we also have to account for ssDNA/CsrA-complexes. Since we have not derived an effective coupled degradation for this complex yet, we explicitly account for its formation and disassembly. This means that we have to include two terms that account for the decrease of free CsrA due to the formation of ssDNA/CsrA-complexes and the increase of free CsrA when such a complex disassembles. Altogether, the differential equation for CsrA reads

$$\partial_t A = \alpha_A - \delta_A A - k_M M \cdot A - k_S A \cdot S - k_D^+ D \cdot A + k_D^- C_{DA}, \tag{14}$$

where C_{DA} is the abundance of ssDNA/CsrA-complexes, and $k_D^{\pm}$ the complex binding and disassembly rate, and the terms containing the novel regulator ssDNA are highlighted in red. Note that we consider the ssDNA to have only one binding site for CsrA in our model. We account for the second binding site analogously to the many binding sites of the sRNA, that is by assuming D to be an effective, single binding site ssDNA, with an effective production rate fitted to experimental data.

The two ssDNA terms highlighted in red also appear in the differential equation for D, since the formation and disassembly of ssDNA/CsrA-complexes in- and decreases also the abundances of ssDNA. The spontaneous degradation is accounted for by a separate degradation term, already

known from the differential equations of the other components. The production term of ssDNA is proportional to P_{COL}, the number of ColicinE2 plasmids in the cell, since ssDNA is an intermediate of the ColicinE2 plasmid replication. The differential equation for ssDNA therefore reads

$$\partial_t D = \alpha_D P_{COL} - \delta_D D - k_D^+ D \cdot A + k_D^- C_{DA}. \tag{15}$$

We are still left with the dynamics of the ssDNA-CsrA-complexes, C_{DA}. The "production" term of the ssDNA/CsrA-complexes is the binding term already known from eqs. (15) and (14), but in this case with a positive sign. The number of complexes is reduced by complex disassembly, which is accounted for by the term $k_D^- C_{DA}$ that also appears in eqs. (15) and (14) with a different sign. Apart from complex disassembly, the complexes can be degraded (in the sense that the complexes and their components are destroyed) spontaneously, which is given by a spontaneous degradation term. Taken together, the differential equation for ssDNA reads

$$\partial_t C_{DA} = k_D^+ D \cdot A - k_D^- C_{DA} - \delta_{C_{DA}} C_{DA}. \tag{16}$$

Effective coupled degradation of ssDNA and CsrA: In the discussion of the ssDNA properties, we saw that ssDNA also has a Shine-Dalgarno sequence, just as the long mRNA. This suggests that we can make the same assumptions for the CsrA-ssDNA-complex as we did for the CsrA-long-mRNA-complex. In the following, we proceed analogously to the simplification of the hierarchical three component model (see Ref. [9]), and assume fast dynamics of complexes. Adiabatic elimination ($\partial_t C_{DA} \equiv 0$) yields

$$C_{DA} = \frac{k_D^+ DA}{k_D^- + \delta_{C_{DA}}} = \frac{k_D DA}{\delta_{C_{DA}}}, \tag{17}$$

with the effective binding parameter

$$k_D := \frac{k_D^+ \delta_{C_{DA}}}{k_D^- + \delta_{C_{DA}}}.$$

By inserting eq. (17) into eqs. (14)-(16), we get our final set of differential equations, which includes all four components:

$$\partial_t M = \alpha_M (P_{COL} + P_{REP} - B) - \delta_M M - k_M M \cdot A, \tag{18}$$

$$\partial_t S = \alpha_{S,0} N + \alpha_{S,c} N \cdot A - \delta_S S - k_S S \cdot A, \tag{19}$$

$$\partial_t A = \alpha_A - \delta_A A - k_M M \cdot A - k_S S \cdot A - k_D D \cdot A \tag{20}$$

$$\partial_t D = \alpha_D P_{COL} - \delta_D D - k_D D \cdot A. \tag{21}$$

The new regulative component ssDNA acts in the same fashion as the sRNA by binding CsrA. Compared to the original three component system, eqs. (12)-(14), the extension with ssDNA therefore resulted in a system of equations with the same types of terms (source term, spontaneous degradation, coupled degradation). We use eqs. (18)-(21) to study gene expression dynamics for all three different strains. This is done by adjusting the corresponding values for P_{COL} and P_{REP}, see section 3. Moreover, we investigate the impact of ssDNA on the regulation of ColicinE2 production and release.

3 Parameter values

For the parameters associated with long mRNA, CsrA and the effective sRNA, we adjusted the values that we determined in our previous study ([9]) according to new measurements. In particular, they were chosen such that they are in accordance with our own experimental measurements (k_M and k_S) or other studies (see below). In particular, the rates read (given per *E. coli* cell volume, and using the shorthand notation "#" for molecule numbers):

rate const.	value	unit	description
α_M	0.05	min^{-1}	production of long mRNA
$\alpha_{S,0}N$	0.1	min^{-1}	baseline production of eff. sRNA
$\alpha_{S,c}N$	0.07	$\text{min}^{-1}\cdot\#^{-1}$	production factor of eff. sRNA
α_A	4.5	min^{-1}	production of CsrA
δ_M	0.04	$\text{min}^{-1}\cdot\#^{-1}$	degradation of long mRNA
δ_S	0.023	$\text{min}^{-1}\cdot\#^{-1}$	degradation of effective sRNA
δ_A	0.00007	$\text{min}^{-1}\cdot\#^{-1}$	degradation of CsrA
k_M	0.007	$\text{min}^{-1}\cdot\#^{-2}$	eff. binding of CsrA to long mRNA
k_S	0.011	$\text{min}^{-1}\cdot\#^{-2}$	eff. binding of CsrA to sRNA

The three degradation rates ($\delta_M, \delta_S, \delta_A$) were determined in previous, experimental studies [5, 7]. The production rates ($\alpha_A, \alpha_{S,c}, \alpha_M$) were fitted such that they reproduce component abundances from experimental studies [7]. The baseline production for the sRNAs, $\alpha_{S,0}N$, is set to a low value, as only few sRNAs are produced in the absence of CsrA [5].

In Ref. [9] we showed that a Poisson-distributed plasmid copy number gives very similar results to a fixed plasmid copy number. This is due to the fact that plasmid replication happens on larger timescales than the regulatory interactions considered in our model. We retain this simplifying

assumption, and set the number of ColicinE2 plasmids constant at $P_{COL} = 20$, which is the average value [8]. For the reporter plasmids in the C_{REP1}, C_{REP2}, S_{REP1} and S_{REP2} strains, we take for type 1 the average copy number $P_{REP} = 55$ [16], and for type 2 the average copy number $P_{REP} = 13$, which we both also assume constant.

Adding ssDNA dynamics to the system introduces three new effective rates, α_D, δ_D, and k_D. We assume that the ssDNA and the mRNA are equally stable, and therefore use the same degradation rate constants for both:

$$\delta_D \equiv \delta_M = 0.04 \text{ min}^{-1} \cdot \#^{-1}.$$

In combination with the K_D-value measurements for ssDNA we could determine the coupled degradation constant to

$$k_D = 0.0001 \text{ min}^{-1} \cdot \#^{-2}.$$

Finally, we have to define the value for the production rate constant of ssDNA, α_D, which has not been explicitly measured yet. However, our experimental data suggests that ssDNA accumulates abundances about an order of magnitude larger than long mRNA. From fitting the ssDNA production to this rough abundance relation, and also to measured delay-times, we obtain

$$\alpha_D = 7 \text{ min}^{-1} \cdot \text{plasmid}^{-1}.$$

To study the influence of ssDNA on the *cea-cel* delay, we varied the value of α_D between 0 and 9, see Fig. S6 and S7. For the validation of our model, we also tested various values of $\alpha_{S,c}$ and α_M (data not shown). These tests showed that, in general, the model is robust to parameter variations, in the sense that changing a parameter value by a few percent only had minor consequences for the resulting delay times and component abundances.

4 Simulation results

The differential equations eqs. (18)-(21) give, in combination with the SOS response model, eqs. (1)-(7) (see [2]), a description of the regulatory interactions governing ColicinE2 production. They enable us to study steady states and the deterministic dynamics of gene expression for all five strains. However, the SOS response [2] shows an inherent stochasticity: The ColicinE2 promoter is not activated permanently during an SOS signal,

but in stochastically appearing bursts of activity. Moreover, most of the regulatory components like long mRNA occur in low abundances, such that also intrinsic demographic fluctuations in the ColicinE2 regulatory system become important. We can study stochastic effects like these by formulating the deterministic dynamics described in eqs. (1)-(7) and eqs. (18)-(21) as a stochastic process. To this end, we consider each component (M, S, A and D, as well as the components of the SOS response system) as random variables that are changed by stochastic events like production or degradation of molecules. Each of these events occurs at an average rate that equals the corresponding term in the mass action model. For instance, the effective coupled degradation of a long mRNA and CsrA happens at a rate $k_M M \cdot A$ (see eq. (18)), which decreases both the abundance of long mRNA (M) and the abundance of CsrA (A) by 1. By defining all remaining stochastic production, degradation and binding events in the system this way, we obtain a description of the SOS response and ColicinE2 regulatory system as a stochastic (Markov) process. We then use the Gillespie algorithm [17] to implement the stochastic process as a stochastic simulation. This simulation enables us to produce stochastically correct realisations of the temporal evolution of the system's random variables. The results from sufficiently large ensembles of these realisations is then the basis for the validation of the theory by experimental data.

In our simulations, we followed the scheme already developed in Ref. [9]: We initiate the system in a non-SOS state, where the parameter c_p in eq. (6) of the SOS response system (see also Ref. [2]) is set to 0, that is, RecA does not cleave LexA. Therefore, B, the number of unrepressed promoters, is low (1 for S_{REP2}, 2 for C_{WT}, 3 for C_{REP2}, 4 for S_{REP1}, and 5 for C_{REP1}). After 200 minutes, we mimic the effect of an SOS signal by increasing the parameter c_p, such that RecA catalyses the degradation of LexA, which acts as repressor for the ColicinE2 operon. This has the effect that the production of both long and short mRNA immediately increases. The SOS signal is stopped again at $t = 500$ minutes. For each set of parameters, this scheme is repeated 2000 times in order to obtain an ensemble of 2000 realisations.

To be able to compare these simulation results with experiments, we have to give an appropriate definition of the *cea-cel* delay in the simulations. As the beginning of "*cea* expression", we define the point in time at which the short mRNA level rises to two times its value before the SOS signal started. Our simulations show that the CsrA abundance decreases during the SOS signal, as more CsrA-sequestering long mRNA is produced. Once there is no free CsrA left, we find free long mRNA in the system. We

define the first point in time at which more than 8 free long mRNAs exist in the system as "*cel* expression". This definition accounts for the fact that in general fewer lysis proteins are produced than toxin proteins [8]. In the experiment, the expression of *cea* and *cel* are defined by the point in time the respective fluorescence intensity reaches five times its basal (i.e. pre-SOS) level. Therefore, the expression times are determined by the appearance of proteins in the experiment, but by the appearance of mRNAs in the stochastic simulations. We choose the different definition of the delay in the simulations, as the specific biochemical rates of many processes involving the mRNAs and proteins are largely unknown, and have to be fitted according to observed abundances. If we included the translation of short and long mRNA to toxin, lysis and fluorescence proteins used in the experimental study into our model as well, we would add several new parameters that require fitting to our mathematical model, without getting a more precise definition of the thresholds that determine the delay. Moreover, comparing a delay in the production of mRNAs with a delay in the production of proteins is valid in our case, since both fluorescence proteins have very similar maturation times [6], and since we are interested in the relative rather than the absolute times of protein expression.

For the C_{WT} strain, we cannot compare the *cea-cel* delay-time from our simulations with experimental results due to the lack of reporter plasmids (see main text). To still be able to validate our stochastic simulations with experimental data in this case, we use the time between the beginning of the SOS response and cell lysis (referred to as "lysis time"), which we also measured in experiments (see Fig. S8). The absolute values of the lysis time will differ between our simulations and experiments, as the simulations do not account for maturation times and other processes of equal duration in all three strains. Therefore, we do not compare lysis times themselves, but the differences of the C_{REP1} and C_{WT} strain's lysis times, which eliminates these constant factors.

In the following, we discuss the results of our stochastic simulations for the experiments presented in the main text.

4.1 The role of CsrA

As a first step to validate the mathematical model for the S_{REP1} strain (that is, with no ssDNA in the system), we test the role of CsrA for the *cea-cel* delay. The corresponding experiment varied the binding affinity of CsrA to long mRNA, see Fig. 2E in the main text. Specifically, the experiment measured the average *cea-cel* delay-time for the original S_{REP1} strain, and

for two mutant strains (CsrA1 and CsrA2) with higher and lower CsrA binding affinity, respectively. The results of these experiments (see Fig. 2E) showed that an increased CsrA binding affinity leads to longer average *cea-cel* delay-times, since the regulation of long mRNA by CsrA sequestration happens more effectively with stronger CsrA binding. Consequently, we found a much shorter average *cea-cel* delay for the mutant with lower binding affinity. In our mathematical model defined by eqs. (18)-(21), the binding affinity of CsrA for long mRNA relates to the parameter k_M^+, which appears in the effective coupled degradation parameter, $k_M = \frac{k_M^+ \delta_{C_{MA}}}{k_M^- + \delta_{C_{MA}}}$ (see Ref. [9] for details on this equation). However, the experiments do not measure the rates k_M^+ and k_M^-, but their ratio k_M^-/k_M^+, known as K_D value (see Fig. 2D of the main text). Since the degradation rate of the complexes is much lower than the dissociation rate (this follows from the fast complex equilibration assumption), we can determine k_M from the complex degradation and the K_D values:

$$k_M = \frac{k_M^+ \delta_{C_{MA}}}{k_M^- + \delta_{C_{MA}}} \approx \frac{k_M^+ \delta_{C_{MA}}}{k_M^-} = \frac{\delta_{C_{MA}}}{K_D} \tag{22}$$

We used eq. (22) and K_D values given in Fig. 2D to determine the binding parameter constants k_M for the S_{REP1} strain and its two mutants CsrA1 and CsrA2. Using these results, we then performed a set of numerical simulations, and recorded the *cea-cel* delay-times. The mean delay times for different values of k_M are shown in main text Fig. 2F, where we also give the parameter values. For the *cea-cel* delay-times we find the same behaviour as in the experiments: higher values of k_M result in broader delay time distributions with a larger average delay time, whereas smaller values give a narrow delay time distribution with an average delay close to the start of the SOS signal. Our results show that the binding of CsrA to long mRNA has a key influence on the delay time distribution. This highlights the critical role of CsrA for the delay between toxin production and release. Therefore, we expect regulative components affecting the abundance of CsrA to have an indirect effect on the duration of the *cea-cel* delay.

4.2 *cea-cel* delay in the five different strains

The experiments discussed in the main text show that the C_{REP1},C_{REP2}, C_{WT}, S_{REP1} and S_{REP2} all have very different *cea-cel* delay times: The C_{REP1} strain, for instance, lyses almost immediately after *cea* expression, whereas the S_{REP1} strain shows a significant *cea-cel* delay. In this section, we employ

stochastic simulations of the model described in eqs. (18)-(21) to study the origin of this difference and the effects of ssDNA production. Moreover, we infer the *cea-cel* delay in the C_{WT} strain from this analysis, which cannot be measured directly in experiments. To this end, we modelled the five strains in our simulations by setting P_{COL} and P_{REP} to the corresponding values (see Table S5): $P_{COL} = 20$ and $P_{REP} = 0$ for C_{WT}, $P_{COL} = 20$ and $P_{REP} = 55$ for C_{REP1}, $P_{COL} = 20$ and $P_{REP} = 13$ for C_{REP2}, $P_{COL} = 0$ and $P_{REP} = 55$ for the S_{REP1} strain, and $P_{COL} = 0$ and $P_{REP} = 13$ for the S_{REP2} strain. For each strain, we simulated 2000 realisations for different ssDNA production rates (α_D) to investigate the role of this novel regulatory component. The results of these simulations are depicted in the form of *cea-cel* delay-time histograms in Fig. S6, in which we, for a clear and concise discussion, only depict the results for the C_{REP1}, S_{REP1}, and C_{WT} strains.

As we showed in section 1, only two factors can in principle be responsible for the different *cea-cel* delays: The total plasmid copy number, and the production of ssDNA. To separately study the influence of total plasmid copy number in the strains, we first analyse the case of no ssDNA production ($\alpha_D = 0$). The delay-time histograms of the three strains for this case are depicted in the first column of Fig. S6. Comparing the histograms, we find that the total plasmid copy number has a significant effect: The *cea-cel* delay distribution of the C_{REP1} strain (75 plasmids in total) has a shorter tail and a more pronounced peak at short lysis times compared to the distribution of the S_{REP1} strain (55 plasmids in total), and the C_{WT} strain (with only 20 plasmids) shows almost no lysis at all. The average delay-time of the S_{REP1} strain (68 minutes) is in good agreement with experimental values (see Fig. 2E). For the other strains, however, the results do not match: The C_{REP1} strain has a mean delay time of 24.1minutes, which is significantly larger than the value we find in our experiments. Our experiments also find that the wild-type indeed does lyse after SOS responses (see Fig. S8), while the histogram of C_{WT} predicts no lysis. These results for $\alpha_D = 0$ show that the plasmid copy number does not suffice to explain the quantitative and (for the wild-type) qualitative behaviour found in our experiments. However, it already accounts for significant differences in the *cea-cel* delay-time distributions between the strains.

Before we study the additional effects of ssDNA, we discuss the origin of these differences in the *cea-cel* delay-time distributions. To this end, we consider the time evolution of the average levels of free CsrA and long mRNA, which are depicted for $\alpha_D = 0$ in the first column of Fig. S7. We find for all three strains that, after the initial equilibration, the average number of CsrA molecules remains at a constant value before an SOS

response ($t < 200$ min), which results from the interactions of CsrA with all its binding partners. Before an SOS signal, the three strains exhibit roughly the same average CsrA level. This changes after the response to an SOS signal (200 min $< t < 500$ min), which reduces the average CsrA level in all three strains: The C_{REP1} strain, containing 75 plasmids, has the lowest CsrA levels, whereas the wild-type strain with only 20 plasmids has a significantly higher level.

Comparing the CsrA and long mRNA levels with the corresponding *cea-cel* delay-time distributions in Fig. S6 shows that the different *cea-cel* delay time distributions are correlated with the average levels of free CsrA (and free long mRNA): The lower the average CsrA level during a SOS signal, the shorter the average *cea-cel* delay-time, and the narrower the delay-time distribution. We can explain this correlation in our mathematical model by the fact that long mRNA production increases in form of stochastic bursts during SOS responses (see section 2). The long mRNAs produced during these bursts must first sequester free CsrA, before their abundance is high enough to produce lysis proteins from it. For the C_{WT} strain, the CsrA level during the SOS response is too high to be sequestered enough by stochastic long mRNA bursts (see Fig. S7 and Fig. S6). For the S_{REP1} and the C_{REP1} strain, however, CsrA levels reach a sufficiently low average abundance during an SOS response that stochastic bursts of free long mRNA are possible, and eventually lysis protein is produced. The average level of free CsrA therefore determines the probability and hence the timing of lysis.

In Fig. S7 we can also see that the single trajectories of CsrA abundance differ qualitatively between the three strains: The trajectories of the C_{REP1} strain show large and abrupt deviations from the mean value, whereas the abundance of CsrA is closer to the mean in the C_{WT} strain. The reason for this difference is the plasmid copy number in each strain: The more plasmids with LexA-regulated promoters, the more CsrA-sequestering elements are produced during an SOS response, and thus the more susceptible the system will be to stochastic bursts in the SOS response, increasing the probability of lysis. We already discussed in the previous paragraph that the number of plasmids also strongly affects the average level of free CsrA, as more plasmids cause a larger average number of promoters to be unrepressed. This effect is due to the fact that the repressor of the ColicinE2 operon, LexA, stochastically binds to and dissociates from the promoter, and thus triggers long mRNA production for short times even in the absence of an SOS signal. The more plasmids present, the larger the number of (transiently) derepressed promoters, and hence the more CsrA-sequestering long mRNA the cell contains. These relations explain

the differences in the *cea-cel* delay between the three strains.

Finally, in order to characterise the additional effect of ssDNA, we consider the plots with ssDNA production (that is, with $\alpha_D > 0$) in Fig. S6. As the ssDNA production rate α_D increases from 0, the average delay times in the C_{REP1} and C_{WT} strain decrease, and also several cells in the C_{WT} strain lyse (see the C_{WT} histogram for $\alpha_D = 1$ in Fig. S6). We attribute this to the fact that increasing α_D results in lower average CsrA levels in the C_{REP1} and C_{WT} strain, see Fig. S7. Consequently, the average *cea-cel* delay-times in Fig. S6 decrease, and lysis of C_{WT} bacteria becomes possible. The S_{REP1} strain, which contains no ssDNA-producing ColicinE2 plasmid, but only reporter plasmids, is not affected by the increase of this parameter. The experimentally observed difference in mean delay times of the C_{REP1} and S_{REP1} strain occur when the ssDNA production rate reaches $\alpha_D = 7$ (see Fig. S6). At this rate, also the C_{WT} shows a broad *cea-cel* delay distribution. For the C_{WT} strain, we cannot compare the average *cea-cel* delay-time from our simulations with experimental results, but have to use the lysis time. For $\alpha_D = 7$, this difference is in the same order of magnitude as the experimental results (see Fig. S8). If the ssDNA production rate becomes too high, large fractions of the cell ensemble lyse even in the absence of an SOS signal, which is not seen for the C_{REP1} strain in experiments.

Taken together, these results show that the additional sequestration of CsrA by ssDNA is required for cell lysis in the C_{WT} strain, and hence necessary to produce the experimentally observed *cea-cel* delays. Therefore, ssDNA plays a key role in the regulation of ColicinE2 release.

4.3 sRNA knock-out mutants

In the main text we also discuss experiments with different sRNA knock-out mutant strains, see Fig. S4. While the two single knock-out cases (no CsrB or no CsrC) are automatically accounted for by the effective sRNA (see the bullet points on sRNA in section 2), the special case of the double sRNA knock-out mutant corresponds to setting $\alpha_{S,0} \equiv \alpha_{S,c} \equiv 0$ in eq. (19) of our model. This means that no sRNA would be produced, which is the main CsrA-sequestering element in our mathematical model. Therefore, our model predicts a large abundance of free CsrA for the double sRNA knock-out case, and hence a significantly larger *cea-cel* delay. However, we do not see this behaviour in our experiments (see Figs. S2 and S4), which in contrast show shorter average *cea-cel* delay-times in the double knock-out mutant. These experimental results indicate that, in the absence of the two sRNAs, yet unknown regulatory mechanisms become

important. In the derivation of our mathematical model (see section 2), we eliminated any subordinate targets for CsrA, and focussed on the main CsrA-sequestering elements in *E. coli*, the sRNAs CsrB and CsrC [7]. Hence, adjusting our model for the double sRNA knock-out mutant would first require to experimentally investigate the detailed interactions and components of the yet unknown regulatory mechanisms, and then to replace the S component and its interactions correspondingly in the model. As the double knock-out mutant is not part of our investigation of the *cea-cel* delay-time in the main text, we do not further extend our model for this very special case. In all other strains and mutants discussed in the main text and the Supplementary Information, sRNAs are produced and also are the main CsrA regulator. Therefore, the aforementioned differences between theoretical model and experimental observations that arise with the double sRNA knock-out mutant do not affect any statements we derive for the single knock-out or original strains using our model, eqs. (18)-(21).

References

1. Janion, C. Inducible SOS Response System of DNA Repair and Mutagenesis in Escherichia coli. *Int. J. Biol. Sci.* **4,** 338–344 (2008).

2. Shimoni, Y., Altuvia, S., Margalit, H. & Biham, O. Stochastic Analysis of the SOS Response in Escherichia coli. *PLoS One* **4** (ed Isalan, M.) doi:10.1371/journal.pone.0005363 (2009).

3. Yang, T. Y., Sung, Y. M., Lei, G. S., Romeo, T. & Chak, K. F. Post-transcriptional repression of the cel gene of the ColE7 operon by the RNA-binding protein CsrA of Escherichia coli. *Nucleic Acids Res.* **38,** 3936–3951 (2010).

4. Timmermans, J. & Van Melderen, L. Post-transcriptional global regulation by CsrA in bacteria. *Cell. Mol. Life Sci.* **67,** 2897–2908 (2010).

5. Weilbacher, T. *et al.* A novel sRNA component of the carbon storage regulatory system of Escherichia coli. *Mol. Microbiol.* **48,** 657–670 (2003).

6. Mader, A. *et al.* Amount of Colicin Release in Escherichia coli Is Regulated by Lysis Gene Expression of the Colicin E2 Operon. *PLoS One* **10.** doi:10.1371/journal.pone.0119124 (2015).

7. Gudapaty, S. *et al.* Regulatory Interactions of Csr Components : the RNA Binding Protein CsrA Activates csrB Transcription in Escherichia coli Regulatory Interactions of Csr Components : the RNA Binding Protein CsrA Activates csrB Transcription in Escherichia coli. *J. Bacteriol.* doi:10.1128/JB.183.20.6017 (2001).

8. Cascales, E. *et al.* Colicin biology. *Microbiol. Mol. Biol. Rev.* **71,** 158–229 (2007).

9. Lechner, M., Schwarz, M., Opitz, M. & Frey, E. Hierarchical Post-transcriptional Regulation of Colicin E2 Expression in Escherichia coli. *PLOS Comput. Biol.* **12** (ed Igoshin, O. A.) e1005243 (2016).

10. Ronen, M., Rosenberg, R., Shraiman, B. I. & Alon, U. Assigning numbers to the arrows: parameterizing a gene regulation network by using accurate expression kinetics. *Proc. Natl. Acad. Sci. U. S. A.* **99,** 10555–10560 (2002).

11. Krishna, S., Maslov, S. & Sneppen, K. UV-Induced Mutagenesis in Escherichia coli SOS Response: A Quantitative Model. *PLoS Comput. Biol.* **3.** doi:10.1371/journal.pcbi.0030041. arXiv: 0701013 [q-bio] (2007).

12. Legewie, S., Dienst, D., Wilde, A., Herzel, H. & Axmann, I. M. Small RNAs establish delays and temporal thresholds in gene expression. *Biophys. J.* **95,** 3232–3238 (2008).

13. Levine, E. & Hwa, T. Small RNAs establish gene expression thresholds. *Curr. Opin. Microbiol.* **11,** 574–579 (2008).

14. Edwards, A. N. *et al.* Circuitry Linking the Csr and Stringent Response Global Regulatory Systems. *Mol Microbiol* **80,** 1561–1580 (2011).

15. Yakhnin, H. *et al.* Complex regulation of the global regulatory gene csrA: CsrA- mediated translational repression, transcription from five promoters by Eσ70 and EσS , and indirect transcriptional activation by CsrA. *Mol. Microbiol.* **81,** 689–704 (2012).

16. Megerle, J. A., Fritz, G., Gerland, U., Jung, K. & Ra, J. O. Timing and Dynamics of Single Cell Gene Expression in the Arabinose Utilization System. *Biophys. J.* **95.** doi:10.1529/biophysj.107.127191 (2008).

17. Gillespie, D. T. Exact stochastic simulation of coupled chemical reactions. *J. Phys. Chem.* **81,** 2340–2361 (1977).
